# Fine-scale contemporary recombination variation and its fitness consequences in adaptively diverging stickleback fish

**DOI:** 10.1038/s41559-024-02434-4

**Published:** 2024-06-05

**Authors:** Vrinda Venu, Enni Harjunmaa, Andreea Dreau, Shannon Brady, Devin Absher, David M. Kingsley, Felicity C. Jones

**Affiliations:** 1 Friedrich Miescher Laboratory of the Max Planck Society, Tübingen, Germany; 2 Deptartment of Developmental Biology, Stanford University School of Medicine, Stanford, CA USA; 3 HudsonAlpha Institute for Biotechnology, Huntsville, AL USA; 4 Howard Hughes Medical Institute, Chevy Chase, MD USA; 5 Present Address: Los Alamos National Laboratory, New Mexico, NM USA; 6 Present Address: CeGAT GmbH, Tübingen, Germany; 7 Present Address: Evotec SE ‘Campus Curie’, Toulouse, France; 8 Present Address: Groningen Institute for Evolutionary Life Sciences (GELIFES), University of Groningen, Groningen, the Netherlands

**Keywords:** Evolutionary genetics, Genomics, Population genetics

## Abstract

Despite deep evolutionary conservation, recombination rates vary greatly across the genome and among individuals, sexes and populations. Yet the impact of this variation on adaptively diverging populations is not well understood. Here we characterized fine-scale recombination landscapes in an adaptively divergent pair of marine and freshwater populations of threespine stickleback from River Tyne, Scotland. Through whole-genome sequencing of large nuclear families, we identified the genomic locations of almost 50,000 crossovers and built recombination maps for marine, freshwater and hybrid individuals at a resolution of 3.8 kb. We used these maps to quantify the factors driving variation in recombination rates. We found strong heterochiasmy between sexes but also differences in recombination rates among ecotypes. Hybrids showed evidence of significant recombination suppression in overall map length and in individual loci. Recombination rates were lower not only within individual marine–freshwater-adaptive loci, but also between loci on the same chromosome, suggesting selection on linked gene ‘cassettes’. Through temporal sampling along a natural hybrid zone, we found that recombinants showed traits associated with reduced fitness. Our results support predictions that divergence in *cis*-acting recombination modifiers, whose functions are disrupted in hybrids, may play an important role in maintaining differences among adaptively diverging populations.

## Abstract (web summary)

The authors build recombination maps of marine, freshwater and hybrid sticklebacks at a scale of 3.8 kb, examining differences in recombination rates and evolutionary implications in populations undergoing adaptive divergence. They find evidence of recombination suppression in hybrids and reduced fitness of recombinants in a natural hybrid zone.

## Main

Meiotic recombination is a key source of genetic diversity shaping the genome and the evolutionary process. By shuffling parental alleles to produce novel haplotypes, recombination impacts how selection acts on linked polymorphisms, increasing its efficacy and facilitating population adaptation. Recombination is essential for proper chromosomal segregation during meiosis in eukaryotes. Defects in recombination can have serious consequences such as inviable gametes, birth defects and developmental abnormalities^1,2^. Together with deep evolutionary conservation of its protein components across all domains of life, recombination is therefore thought to be highly constrained^3^.

Despite these constraints, recombination varies greatly: significant and heritable differences in recombination location and rate have been observed among individuals^4,5^, sexes^6–9^ and species^10–12^. Across the genome, recombination rate can vary by orders of magnitude^13,14^—in some species involving ‘hotspots’ and ‘coldspots’^4,15–17^. Understanding the regulation of crossover location and frequency is thus a core objective of recombination studies. Evidence suggests that a hierarchical combination of *cis*-acting factors including chromatin state, epigenetic factors, and DNA sequence context are important^18–21^. Additionally, *trans*-acting factors can also modify recombination rate genome wide (for example, RNF212 (refs. ^22–24^)) or locally (for example, PRDM9 (refs. ^25–27^)).

Genomic variation in recombination causes uneven shuffling across the genome, inevitably shaping the distribution of genetic variation visible to natural selection, and potentially placing recombination modifiers themselves under selection^28–30^. Adaptation in natural populations typically involves multiple, often clustered, loci^31–35^. This dispersed but non-random distribution implies uneven linkage between adaptive loci and raises further questions: if recombination is essential in meiosis, what are the functional molecular constraints? What are the effects of shuffling alleles among adaptive loci? How do selective forces shape recombination variation?

Threespine stickleback fish offer an attractive vertebrate system to investigate how molecular constraints and evolutionary forces shape recombination in nature. First, a number of marine–freshwater hybrid zones exist, in which hundreds of loci underlying marine versus freshwater adaptation are well characterized at kilobase resolution^32,36^. Second, crosses with large clutch sizes can be raised to build individual recombination maps. This is key because direct detection of parental crossover events from pedigrees is not sex averaged nor confounded by population demography and selection. It thereby provides an accurate snapshot of the contemporary recombination landscape that is complementary to widely used linkage disequilibrium (LD)-based estimates. Third, sticklebacks benefit from a mature genomic and molecular toolkit, including a high-quality, well-annotated genome assembly with variation and functional data. Finally, we can monitor allele frequencies in natural populations over time, especially in recombinant fish, providing powerful insight to the fitness effects of recombination in the wild.

Following the Pleistocene glacial retreat from the Northern Hemisphere (10–20,000 years ago) ancestral marine sticklebacks colonized and adapted to newly formed freshwater lakes and rivers through parallel changes in numerous morphological, physiological and behavioural traits^37^. This rapid adaptive marine–freshwater divergence draws on standing genetic variation at more than 242 loci across the genome (false discovery rate, FDR of 0.05 (ref. ^32^)) via ongoing gene exchange through hybrid zones^38,39^.

In such populations experiencing both high gene flow and strong divergent selection pressures, recombination may be beneficial and/or deleterious: it can introduce beneficial alleles into a population and yet may counteract adaptive divergence by homogenizing differences. Under this scenario, natural selection may theoretically favour linkage among alleles as ‘adaptive cassettes’^40,41^ and recombination modifiers with underdominant modes of action (where recombination suppression occurs only in hybrids) may be particularly favoured. Further, increased variation in recombination itself (for example, heterochiasmy) might be beneficial to (meta-)populations exposed to heterogeneous selection regimes by facilitating adaptation to their local environment while navigating the costs and benefits of gene flow involving beneficial and deleterious alleles from divergently adapted neighbouring populations. In sticklebacks, several studies have confirmed strong heterochiasmy and that adaptive loci fall into regions of low recombination^34,42,43^ but it remains unclear whether recombination rates have become locally modified or how molecular constraints may dictate the formation of adaptive cassettes.

In species lacking PRDM9, local recombination rates have been assumed to be highly conserved^44^. However, although sticklebacks lack a functional PRDM9, their recombination hotspots vary considerably between adaptively diverged populations^45^. Neither the fitness consequences nor the molecular mechanisms of this variation have been addressed at high resolution. In this Article, we contrast high-resolution genomic recombination maps of marine, freshwater and hybrid ecotypes, intersect them with genomic and molecular features associated with crossovers, and explore the potential fitness implications of recombination among linked adaptive loci in admixed individuals from a hybrid zone. Combined, our results provide a unique platform for exploring the interplay of recombination and natural selection in adaptively diverging populations.

## Results

### Female-biased heterochiasmy in individual recombination maps

**Fig. 1:**
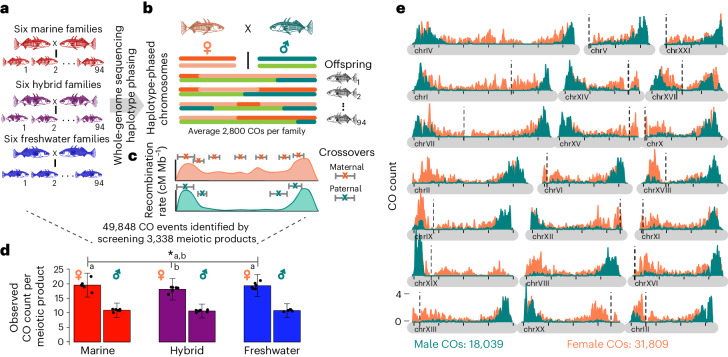
Construction of single-individual recombination maps by nuclear family whole-genome sequencing. **a**, Eighteen large-clutch nuclear families generated by in vitro fertilization of fish collected from the River Tyne. Six ♀MAR×♂MAR families (red), six ♀FRESH×♂FRESH families (blue), three F1-hybrid families from ♀MAR×♂FRESH F1 hybrids and three reciprocal F1-hybrid families (purple).MAR, marine; FRESH, freshwater. In each family two parents and 86–94 offspring (median 93) are whole-genome sequenced to more than 38× coverage for parents and >10× coverage for each offspring. **b**, For each nuclear family, paternal and maternal chromosomes of offspring are phased separately. Switches from the first haplotype to the second haplotype (dark to light orange in the maternal chromosome and dark to light green in the paternal chromosome) are recorded as crossover (CO) events; defined as the interval between the last SNP of the first haplotype and the first SNP of the second haplotype. **c**, The distribution of crossover intervals across the chromosome is used to build paternal and maternal recombination maps. **d**, The genome-wide crossover count per meiotic product identified from individuals of each ecotype and sex shows consistently higher crossover count in females than males. Error bars represent standard deviation. Hybrid females (*N* = 6, marked b) have significantly reduced crossover count compared with marine and freshwater female crossover count together (*N* = 12, marked a; two-sided Wilcoxon rank sum *W* = 58 and *P = 0.041). The observed number of crossovers corresponds to a mean sex-averaged genetic map length of 1493cM and a genome-wide average recombination rate of 3.24cM/Mb, broadly consistent with map lengths in previous studies (1,404 centimorgans (cM), range: 993–1,963 cM, from refs. ^9,42,80,91,92^). **e**, The distribution of 31,809 crossovers from 18 females (orange) and 18,039 crossovers from 18 males (green), plotted in 100 kb sliding windows across all 21 chromosomes, shows a more pronounced periphery-biased distribution in males than females. Crossovers overlapping scaffold gap boundaries are excluded. The approximate position of centromeres in all but three chromosomes (chrII, chrIV and chrVIII) are plotted as vertical black dotted lines. The black tick marks along chromosomes indicate 5 Mb intervals. On the 18 chromosomes for which approximate centromere location is known^93^, acrocentric chromosomes tend to concentrate male recombination at the end of the long chromosome arm, except for chrXIX (the sex chromosome) for which males are the heterogametic sex and recombination between X and Y occurs in the PAR.

We built high-resolution, genome-wide maps of recombination crossover locations for each of 36 individuals from wild-derived strains of marine, freshwater and F1-hybrid fish using whole-genome sequencing followed by whole-chromosome haplotype phasing of large nuclear families with 86-–94 offspring (Fig. 1a–c). Offspring of nuclear families carry recombinant chromosomes generated during maternal and paternal gametogenesis, collectively harbouring thousands of recombination crossovers from each parent (>2,800 per family). We use the term crossover to refer specifically to detected recombinant chromosomes transmitted to offspring and chiasmata to refer to crossovers in the parental tetrad. This distinction highlights that crossovers observed in offspring cannot be easily equated to half of the number of chiasmata in parental tetrads (bivalents) since, for a given chiasma, only two of the four chromatids may recombine, and transmission of the resulting four chromatids to subsequent generations can be biased by a number of processes (for example, haploid selection, meiotic drive and selection on zygotes). From 3,338 meiotic products (gametes inferred from haplotype phasing of offspring), we assembled a comprehensive set of 49,838 crossover locations (18,039 paternal and 31,809 maternal) with median resolution of 3,845 bp (Supplementary Fig. 1).

The resulting genetic maps reveal significantly more crossovers in females than males (Fig. 1d; mean map length: 1,906 centimorgans (cM) in females, 1,081 cM in males; mean crossovers per gamete: 19.06 ± 0.34 s.e.m. in females, 10.81 ± 0.09 s.e.m. in males; *P* < 8 × 10^−16^), echoing previous studies^9,46^. On average, only half the 21 paternal chromosomes (50.0%, mean 10.5 chromosomes) and 68.6% of the 21 maternal chromosomes (mean 14.4 chromosomes) inherited by offspring have one or more crossovers. This strength of heterochiasmy (1.76-fold more crossovers per meiosis in females than males) places sticklebacks among outliers of fish species^47^.

At the chromosome level, we continued to observe a stark contrast between maternal and paternal crossovers. First, the mean number of crossovers is positively associated with chromosome size in females, but regardless of size remains stable at 0.5 for males (Supplementary Fig. 2). Second, the three largest chromosomes (I, IV and VII) that evolved via fusion of ancestral chromosomes (Supplementary Fig. 3), average more than one maternal crossover and also are exceptions in males (deviating above the trend of 0.5 crossovers). Both results can be understood in the context of crossover interference, whereby the presence of one crossover inhibits a second nearby. Indeed, the distribution of inter-crossover distances deviates significantly from that expected if crossovers occurred independently of each other, with male gametic products showing both greater inter-crossover distance and less variation than females (Supplementary Fig. 4). Finally, crossovers lie predominantly in chromosome peripheries, especially in male gametic products (Fig. 1e), with more than 70% of male crossovers occurring within the first or last 15% of the chromosome. Females show a more moderate crossover periphery bias (47%).

To investigate the effects of heterochiasmy on the spread of adaptive loci, we used forward simulations of three linked loci in a metapopulation undergoing adaptive divergence with gene flow. Contrasting strong heterochiasmy versus none, while keeping the overall recombination rate constant, we compared how quickly freshwater-adaptive alleles spread from one freshwater population to another via a marine–freshwater hybrid zone (Supplementary Methods 1). Increased variance in recombination rate due to heterochiasmy has little effect on the spread of adaptive alleles (cf. Supplementary Figs. 9 and 11), but instead helps to maintain significantly greater differentiation across the marine–freshwater hybrid zone (cf. simulation 1 with heterochiasmy to simulation 3 without heterochiasmy; Supplementary Table 8). Contrasting a scenario where both males and females have high recombination rates to one where recombination is suppressed in males, we found the latter marginally increases the probability and speed of a selective sweep and spread of adaptive alleles (cf. simulation 5 to simulation 3; Supplementary Figs. 13 and 11, respectively).

### Genome-wide recombination reduction in hybrid females

While recombination variation between sexes is common^4,7,9,48,49^, studies have also reported variation between closely related species or diverging ecotypes^11,45,50^. At a genome-wide level, we find that marine and freshwater stickleback ecotypes do not differ in overall number of crossovers. Instead, a significant reduction in overall recombination rate is seen in F1-hybrid females, compared with females of marine and freshwater ecotype (mean number of crossovers in F1-hybrid females: 18.169 ± 0.4 s.e.m., marine and freshwater females: 19.5 ± 0.42 s.e.m.; Wilcoxon rank sum test, *W* = 58, *P* = 0.042; Fig. 1d).

### Ecotype and sex effects on recombination rate

**Fig. 2:**
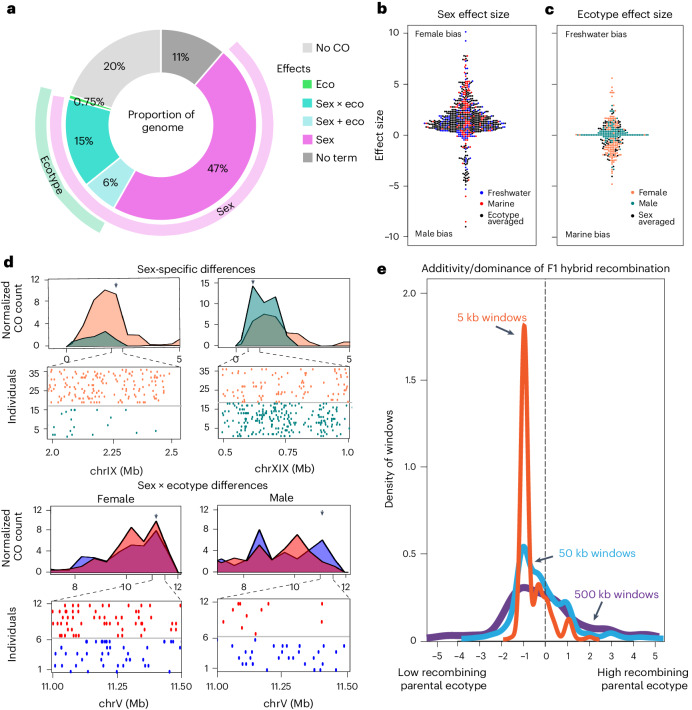
Sex and ecotype explain significant inter-individual variation in local genomic recombination. **a**, The percentage of the genome in which recombination rate variation among individuals can be explained by sex and/or ecotype, determined by linear modelling on recombination rate in 500 kb sliding windows (CO, crossover; eco, ecotype). **b**, The effect size (magnitude of difference in recombination rate) in local genomic windows where sex explains significant differences in recombination crossover count (black, sex effect observed in both ecotypes; red, sex effect only observed in marine ecotypes; blue, sex effect only observed in freshwater ecotypes). **c**, The effect size (magnitude of difference in recombination rate) in local genomic windows where ecotype explains significant differences in recombination crossover count. Marine and freshwater fish tended to have higher recombination rate in roughly the same number of windows (black, ecotype effect observed in both sexes). When sex-specific ecotype differences were observed, freshwater males tended to have higher recombination than marine males (green), whereas marine females tended to have higher recombination than freshwater females (orange). It is notable though, that the maximum ecotype effect size was observed in a genomic window with freshwater female recombination rate being 5.57 cM higher than that of marine females. **d**, Examples windows with significant differences in crossover counts (maximum effect size in plotted windows denoted with arrow). Sex-specific crossover counts (orange, female; green, male) in a 5 Mb region of chrIX (top left) and PAR in chrXIX (top right) are shown, with a zoom in of crossover midpoints below. Bottom: ecotype-specific crossover counts (blue, freshwater; red, marine) in a 5 Mb region of chrV, with a zoom in of a window exhibiting sexually antagonistic bias (marine biased in females, bottom left; freshwater biased in males, bottom right). **e**, Density estimates of dominance/additivity ratio of recombination crossovers in F1 hybrids relative to pure ecotype parental crossovers in genomic windows showing ecotype-divergent recombination. A value of zero indicates the high recombination allele behaves additively in F1s relative to low and high recombining parents, 1 dominantly, −1 recessively and values >1 or <−1 imply over or underdominance of recombination, respectively. F1-hybrid recombination rate is biased towards the low recombining parental ecotype at all three (500 kb, 50 kb and 5 kb) scales of resolution.

To identify local genomic recombination variation undetectable at a genome-wide level, for example, due to a *cis-*acting recombination modifier, we used linear modelling to identify windows (500 kb) in which sex and/or ecotype can explain significant variation in crossovers. Ecotype predicts recombination rate in 6.75% of the genome (~31.25 Mb; Fig. 2a), independent of sex and with no clear bias in the direction or effect size. By contrast, sex explains significant recombination rate variation in 53% of the genome (~245 Mb; Fig. 2a). This effect is independent of ecotype, with females showing higher recombination rate effect sizes (for example, Fig. 2d, top left, up to 10.23 cM), compared with males (effect sizes up to 5.9 cM in autosomes, and 8.87 cM in the pseudo-autosomal region (PAR); Fig. 2b,d, top right). Interestingly, recombination variation among individuals in 15% of windows depends on both sex and ecotype (sex × ecotype; Fig. 2d, bottom). Here, when males differ in recombination, freshwater males have higher recombination rates than marine males in most windows (Fig. 2c, green points)—an effect that cannot be attributed to differences in detection power since nucleotide variation in these regions does not differ significantly between marine and freshwater males. In contrast, when ecotype explains significant variation among marine and freshwater females, the effect is reversed—marine females tend to have higher recombination rate than freshwater females (Fig. 2c, orange points).

### Partially recessive recombination rates in hybrids

Using families with F1-hybrid parents, and considering only genomic windows with significant differences across ecotypes, we asked whether recombination rate behaved in a dominant or additive manner relative to the high- and low-recombining alleles carried by parental marine and freshwater ecotypes. We found F1-hybrid recombination rate to be biased towards matching the low-recombining parental ecotype irrespective of whether that parent was marine or freshwater, and independent of sex and scale of resolution (Fig. 2e and Supplementary Fig. 5). This implies that the higher recombination allele acts recessively and the lower recombination allele acts dominantly. We hypothesize that modifier mechanism(s) increasing local genomic recombination rate in either ecotype behave partially recessively in F1 hybrids and may reflect delays in crossover repair due to allelic mismatches in F1 heterozygotes.

### Fine-scale recombination hotspots and coldspots

**Fig. 3:**
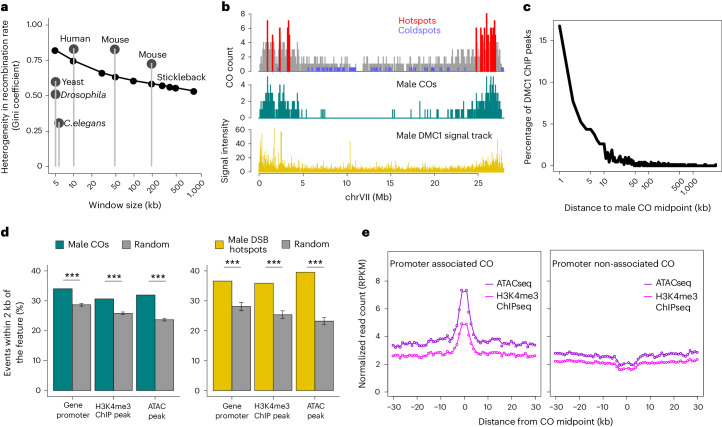
Molecular mechanisms and genomic features shaping the stickleback recombination landscape. **a**, Sticklebacks show high heterogeneity (Gini coefficient) in their genomic crossover distribution, more similar to human^5^ and mouse^4^, and considerably higher than yeast^16^, *Drosophila*^13,94^ and *C.* *elegans*^14^. The grey points show heterogeneity at genomic scales applied in each study and the black points show stickleback heterogeneity coefficients at multiple different scales. **b**, Examples of the crossover landscape (grey peaks) on chromosome VII highlighting recombination hotspots (red peaks) and coldspots (blue peaks) detected using all individuals of marine, freshwater and hybrid ecotype (top). The overall shape of the male crossover landscape (middle, green peaks) is similar to the DSB landscape inferred from DMC1 ChIPseq in males (bottom, yellow peaks). **c**, A total of 38.7% of DMC1 ChIPseq peaks (a proxy for DSB hotspots) have a male crossover within 5 kb distance, despite different individuals being used in the distinct assays. The plot shows distance from DMC1 ChIPseq peaks to the nearest crossover in stickleback males. **d**, In stickleback males, crossovers (green bars, left) and DSB hotspots (yellow bars, right) are significantly associated with gene promoters, and active transcription predicted by chromatin accessibility (assayed with ATACseq) at promoters and the histone modification mark H3K4me3. Grey bars ± s.d. represent the proportion of crossovers (left) and DSB hotspots (right) found within 2 kb of features after shuffling genomic location *N* = 1,000 times. This provides an empirical distribution for testing the significance of the observed proportions of crossovers and DSBs near each feature: ***P < 0.001. **e**, Promoter-associated crossovers coincide with elevated signals of chromatin accessibility (purple) and H3K4me3 (pink, left) consistent with active transcription. In contrast, crossovers further than 2 kb away from the nearest promoter coincide with decreased signals of chromatin accessibility and H3K4me3 (right). Since the crossover and DSB datasets are derived from different individuals, we cannot rule out that these distal crossovers are in fact associated with active transcription in those individuals. RPKM, reads per kilobase million.

The frequency and location of crossovers in sticklebacks generate a significantly non-random recombination landscape (80% of the crossovers occur in less than 35% of the genome), involving both ‘hotspots‘ and ‘coldspots’. The heterogeneity in crossover distribution (Gini coefficient) is intermediate to species that constrain recombination in tight hotspots via the PRDM9 pathway (for example, mouse^4^ and human^5,25^), and species that lack PRDM9 and thus have less intense hotspots (yeast^16^) or none (*Drosophila*^13^ and *Caenorhabditis elegans*^14^; Fig. 3a).

We defined stickleback hotspots as intervals containing multiple crossovers among all 36 parent individuals (*N* = 432 with 5% FDR; Fig. 3b). At fine-scale 5 kb intervals, hotspots show crossover rates 3.7-fold above those in the flanking region and tenfold higher than the genome-wide average (range: 0.15–0.45 cM). Stickleback hotspots are sixfold less intense than mouse and human (0.001–3 cM (ref. ^51^)) and nearly fivefold less abundant (after accounting for genome size^4^). Considering the genomic colocalization of crossovers observed in pools of marine-, freshwater- and hybrid-ecotype fish we identified 12 distinct genomic regions (comprising 31 overlapping 5 kb windows) with significant log_2_-fold change differences in crossover counts between any of the three groups (5% FDR; Supplementary Tables 2–5). There are more freshwater ‘hotspot’ regions than marine or hybrid, with the two strongest ecotype-divergent hotspot windows containing log_2_-fold differences in crossover count between freshwater and hybrid individuals of >6.34 (chrXXI: 11400304–11405304) and >6.34 between marine and hybrids (within PAR region chrXIX: 1625740–1630740). All 31 hotspot windows contain crossovers from 5–12 fish, indicating ‘hotness’ is not driven by a single individual. Most hotspots are exclusively ‘hot’ in only one ecotype and at hotspots differing between marine and freshwater ecotypes, crossover counts of F1 hybrids are closer to the ‘cold’ ecotype (Supplementary Tables 3–5).

We identified 499 distinct recombination coldspots (defined as contiguous regions that should accommodate at least five crossovers under a uniform distribution, but contained none) with a minimum size of 212 kb. While hotspots are most often located in subtelomeric regions of chromosomes, coldspots are most often in the centres or telomeric ends of chromosomes (Fig. 3b). In female F1 hybrids, but not males, coldspots are significantly larger (median 345 kb) than freshwater (323 kb) or marine coldspots (311 kb; Wilcoxon rank sum tests, *W* = 20,423 and 24,869; *P* = 0.027 and 0.023, respectively).

### DSBs and gene features shape recombination

Regulation of crossover location in meiosis may occur via ‘upstream’ mechanisms determining the location of DNA double-strand breaks (DSBs), such as H3K4me3 histone modifications known to affect chromatin accessibility, and via ‘downstream’ mechanisms influencing whether DSB repair resolves via either crossover or non-reciprocal non-crossover^52–54^ pathways. To determine the relative importance of ‘upstream’ versus ‘downstream’ mechanisms in shaping stickleback recombination, we generated and compared a DSB map in male meiotic cells with male crossover maps generated from nuclear families. We raised an antibody against stickleback meiotic protein DMC1, a protein that binds the single-strand ends of meiotic DSBs. We then performed a modified chromatin immunoprecipitation protocol followed by sequencing (anti-stickleback DMC1 ChIPseq) to recover DMC1-bound single-stranded DNA DSB intermediates^55^ in testes tissue, using liver tissue as a negative control.

Testes-specific DMC1 signal is elevated at the chromosome peripheries, in a pattern highly reminiscent of male crossovers (Fig. 3b). We identified 1,090 DMC1 peaks indicative of DSB hotspots (median size 1.4 kb), 72.2% of which are located in the first or last 15% of chromosomes, consistent with the crossover distribution observed in nuclear families (70%). Further, we see prominent enrichment of testes-specific DMC1 peaks close to male crossovers (38.7% of DMC1 peaks have a male crossover within 5 kb; Fig. 3c). This enables a lower estimate that two-fifths of stickleback DSB hotspots are repaired via the crossover pathway, and the rest via a non-crossover resolution pathway. We anticipate this estimate would increase if the same meiotic cells could be used in both assays. The number of crossovers per male meiosis, 10.81, roughly corresponds to at least 21.62 chiasmata, assuming at least one obligate chiasma per homologous chromosome pair and no transmission bias. A recent study using immunolabelling of leptotene RAD51 (ref. ^56^) estimated about 40 DSBs per stickleback male. Combined, these results suggest about half the DSBs are resolved as crossovers and that the genomic recombination landscape in sticklebacks is shaped by mechanisms determining the location of DSB initiation.

We next asked whether specific genomic features are associated with crossovers. In many mammals, the PRDM9 histone methyltransferase (and other *trans*-acting recombination mediators) recognizes specific DNA motifs, while in other species, recombination crossovers coincide with features such as promoters and repetitive regions^19,57^. In stickleback families, male crossovers, but not female crossovers, are significantly associated with genic regions and all genic features (most strongly with promoters, but also introns, exons and more weakly with transcription-end sites). Sticklebacks lack a homologue encoding *PRDM9*, and we find no evidence of motifs enriched in crossover hotspots except short CA/GT or G/C rich motifs that are significantly correlated with hotness (Supplementary Table 6). DMC1 ChIPseq-predicted DSB hotspots from male testes tissue, as well as male crossovers from nuclear families, are proximally associated with regions of active transcription (Fig. 3d). A total of 46.8% of crossovers and 54.2% of predicted DSB hotspots fall within 2 kb of active transcription while the remaining half are more distal. We note with interest that crossovers occurring further than 2 kb from the nearest promoter colocalize with a decrease in chromatin accessibility and H3K4me3 marks (Fig. 3e) suggesting they are enriched in regions of inactive transcription, and ruling out any association of H3K4me3 in a manner echoing species with *PRDM9*. These signatures may indicate an undescribed means of recombination regulation away from gene features in stickleback fish.

### Adaptive ‘islands’ fall in regions of low recombination

**Fig. 4:**
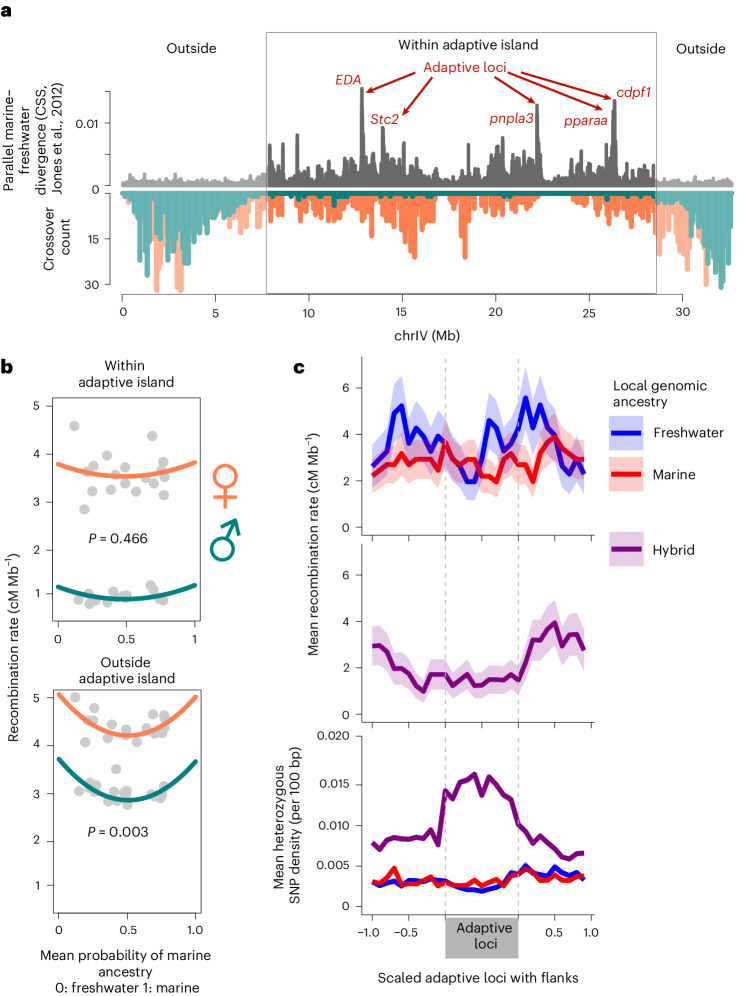
Recombination suppression within and among adaptive loci. **a**, Parallel adaptive divergence (grey, CSS, cluster separation score from Jones et al.^32^) and sex-specific crossover counts in 100 kb sliding windows (male, green; female, orange) show suppression of male crossovers and, to a lesser extent, of female crossovers, within an adaptive island on chromosome IV. **b**, The recombination rate within adaptive islands is lower than outside in both sexes. Recombination suppression outside adaptive islands, but not inside, increases in individuals with more admixed genomes. The significance of this non-linear relationship was tested using a quadratic regression with the squared coefficient significantly greater than 0 (*β*_1_ = 3.325 ± 1.015 s.e.m.) and explaining a significant amount of variation in the model, *F*_1,32_ = 10.738, *P* = 0.003. **c**, Admixed genetic ancestry at focal adaptive loci (heterozygous for marine and freshwater haplotypes, purple) is associated with lower recombination rate within the divergent adaptive haplotypes and elevated recombination rate on the divergent haplotype flanks (top). Mean female recombination rate grouped by their local ancestry across 242 adaptive loci with standard error intervals (top, homozygous marine: red and homozygous freshwater: blue; middle, heterozygous: purple) shows reduced recombination in loci with hybrid ancestry. Mean heterozygous SNP density in 100 bp windows across the scaled adaptive loci in the same categories shows higher heterozygous SNP density in adaptive loci with hybrid ancestry (purple line, bottom) than marine or freshwater ancestry (blue and red lines).

When adaptive divergence evolves in the face of gene flow, recombination shuffling of adaptive loci can lead to mismatched combinations of divergently adaptive alleles and may have important evolutionary implications for individual fitness and population adaptation. Adaptive divergence of marine and freshwater stickleback ecotypes draws strongly on standing variation in the form of highly divergent marine and freshwater haplotypes (up to 4% sequence divergence) at 242 genomic loci (FDR 0.05), including three large inversions^32^. Across the genome, these loci fall in regions of low recombination rate (for example, Fig. 4a; see also refs. ^34,42,43^). Defining adaptive islands as chromosomal regions of linked adaptive alleles, we found recombination rate in males to be nearly threefold lower inside adaptive islands compared with outside (Fig. 4a,b) and in females 1.2-fold lower (Fig. 4a,b). Using whole-genome sequencing data, we calculated the marine genome ancestry proportion in each individual and asked whether recombination rate within and outside ‘adaptive islands’ differs among individuals depending on their genome-wide admixture proportion using a non-linear quadratic regression. Individuals with admixed genome composition tend to have significantly lower recombination rates outside adaptive islands, compared with individuals with either freshwater or marine ancestry (Fig. 4b).

### Recombination-suppressing effects of heterozygosity

Further, individuals heterozygous for marine and freshwater haplotypes at adaptive loci show locally suppressed recombination rates with higher rates immediately beyond the boundaries of divergent adaptive loci (Fig. 4c). Associated with this boundary effect is elevated heterozygosity (mismatches between chromosome homologues) within adaptive loci (Fig. 4c) and lower heterozygosity in the flanks. Our heterozygosity-associated recombination suppression is not confounded by differences in population history or selection like the LD-based estimate *rho*, because recombination is quantified directly from nuclear families not population-level data. The effect is consistent with our previous observations of recombination suppression in inversion heterokaryotypes (Supplementary Fig. 6)^58^ and colocalization of recombination coldspots with high heterozygosity in genomic regions of otherwise high recombination rate (for example, Supplementary Fig. 7a). In fact, the vast majority of crossovers identified occur in regions of low heterozygosity (less than one heterozygous site per 100 bp; Supplementary Fig. 7b). We hypothesize that high heterozygosity slows the rate of homologue engagement during DSB repair and increases the likelihood of repair by a non-crossover pathway^59,60^. This phenomenon may have important implications for populations undergoing adaptive divergence with gene flow—using forward simulations of three linked loci, we found recombination suppression in *cis*(coupled)-heterozygotes increases the rate at which freshwater-adaptive alleles spread through marine–freshwater hybrid zones and are swept to fixation in new freshwater populations (Supplementary Figs. 9–16). Further, under a selection–migration balance, recombination suppression in *cis*(coupled)-heterozygotes helps maintain significantly higher divergence in allele frequencies between marine and freshwater populations (cf. Supplementary Table 8 simulations 1 and 2 and Supplementary Figs. 9 and 10).

### Fitness effects of recombination in natural populations

Quantifying the fitness effects of recombination in naturally evolving populations is notoriously difficult, requiring large numbers of individuals who have recombined among known fitness-affecting loci. Stickleback chromosome IV is an important chromosome harbouring a number of loci underlying variation in fitness^61^, major skeletal traits^36,62^ and other pleiotropic effects^63^, and includes five genomic regions with extraordinarily strong molecular signatures of parallel marine–freshwater divergence (Fig. 3a; *eda* chrIV: 12.81 Mb, *Stc2* chrIV: 13.94 Mb, *pnpla3* chrIV: 22.21 Mb, *pparaa* chrIV: 26.30 Mb and *CDPF1* chrIV: 26.35 Mb)^32^. Although these five loci span a large physical distance (>13.5 Mb), the low recombination rate (1.31 cM Mb^−1^) of this region maintains strong linkage among adaptive alleles facilitating their inheritance as a linked adaptive cassette. Furthermore, the strength of selection acting on this region in newly founded freshwater populations is extremely strong (contemporary evolution estimates of selection at *eda* and its linked genomic loci s* = 0.5 (ref. ^61^)). It stands to reason that when gene flow occurs between divergently adapted populations, the shuffling effects of recombination among adaptive loci may have deleterious consequences for the fitness and survival of admixed offspring, especially considering the importance of *cis*-regulatory control of gene expression divergence in this species^64^.

**Fig. 5:**
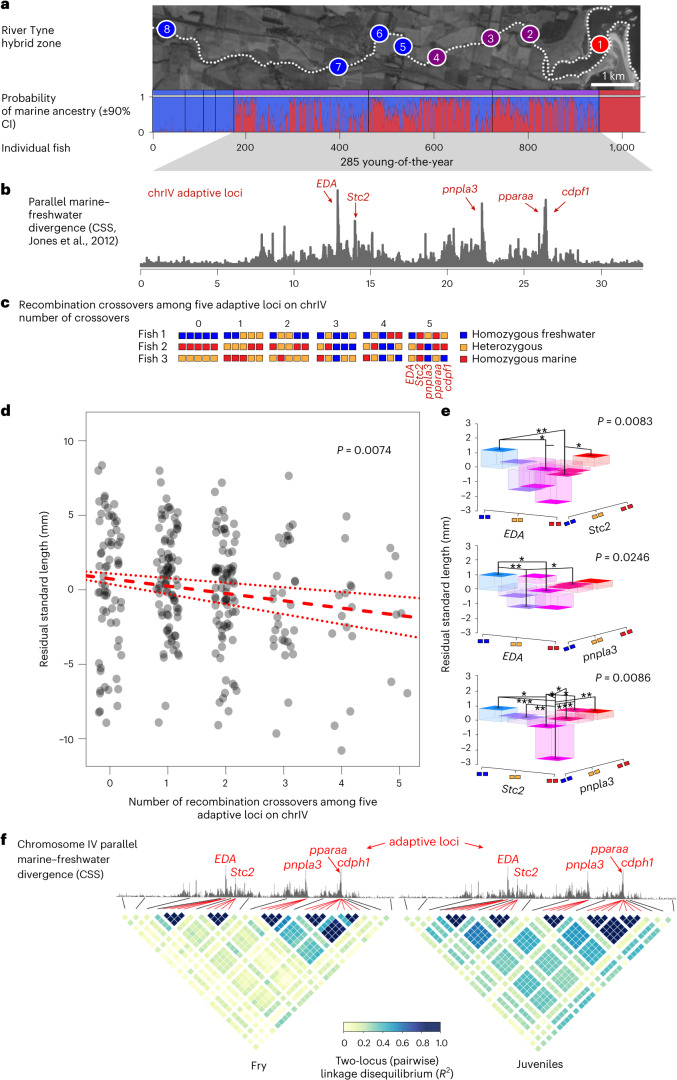
Selection against hybrid recombinants in a natural hybrid zone. **a**, Marine genomic ancestry score of individuals sampled from the River Tyne hybrid zone, Scotland (red, marine; blue, freshwater). **b**, Parallel adaptive divergence between global marine and freshwater populations on chromosome IV (from Jones et al.^32^). Black chromosome tick marks indicate 5 Mb intervals. **c**, Example genotypes giving rise to minimal crossover counts among five adaptive loci on chromosome IV. **d**, Body size (residual standard length relative to cohort mean), a proxy for fitness, significantly decreases as the minimum number of recombination events among adaptive loci increases (linear model; slope *β* = −0.492 ± 0.182 s.e.m.; *F*_1,281_ = 7.251, *P* = 0.007). **e**, Individuals carrying mismatched marine and freshwater alleles across eda:Stc2, eda:pnpla3, Stc2:pnpla3, Stc2:CDPF1 and Stc2:pparaa loci are significantly smaller in size than individuals with non-mismatched allelic combinations. This effect is significant, both as a simple linear model of variation in standard length explained by minimum number of crossover events (slope of −0.492 ± 0.182 s.e.m. mm per recombination event, ANOVA *F*_1,281_ = 7.2506, *P* = 0.0074) (**d**) and under an epistasis model between multi-locus genotypes at pairs of loci (ANOVAs *F*_1,281_(range) = 4.56–7.07, *P*(range) = 0.008–0.034) (**e**). The *P* values next to the bar plot show significance of overall epistasis model, while asterisks indicate the significance of post hoc two-sided *t*-tests *P < 0.05, **P < 0.01 and ***P < 0.001. **f**, LD (*R*^2^) among adaptive loci (red lines) increases (blue colour gradient) as the cohort ages—a pattern consistent with selection against recombinants.

We hypothesized that recombinants carrying mismatched combinations of alleles across these five loci have reduced fitness relative to non-recombined individuals. Leveraging a previously described natural marine–freshwater hybrid zone with large population sizes, we explored potential fitness effects of recombination in the wild^65^ in two ways: first using standard length as a proxy for fitness (ability to grow) and second using changes in within-chromosome two-locus LD over temporal samples of the same cohort as an indication of differential survival of recombinant genotypes. Two hundred eighty-five young-of-the-year from the River Tyne hybrid zone (Fig. 5a)^65^ were fin clipped, tagged, released and genotyped using a 3,084 SNP genome-wide custom genotyping array. Focusing on chromosome IV, we calculated the minimum number of parental recombination events needed to explain an individual’s multi-locus genotype at the five linked adaptive loci (Methods and Fig. 5b,c).

We observed a significant negative relationship between body size and the minimum number of recombination events an individual carries among adaptive loci on chromosome IV (slope of −0.492 ± 0.182 s.e.m. mm per recombination event, *F*_1,281_ = 7.251, *P* = 0.007; Fig. 5d). Recombinant individuals carrying a mixture of marine, freshwater and/or heterozygote genotypes at the five adaptive loci are smaller than individuals with fewer recombination events, suggesting these individuals grow less well than non-recombinants. This cannot be explained by single locus genotypes at the five individual adaptive loci alone (all *P* values >0.05). Rather, consistent with the importance of a linked adaptive cassette, we find evidence for epistasis among adaptive loci on this chromosome; two-locus genotypes at *eda:Stc2*, *eda:pnpla3*, *Stc2:pnpla3*, *Stc2:CDPF1* and *Stc2:pparaa* explained significant amounts of variation in standard length (analysis of variance (ANOVA) *F*_1,281_(range) = 4.56–7.07, *P* = 0.0083–0.0337; Fig. 5e). Individuals with mismatched two-locus genotypes (for example, MM:FF, MM:FM and FM:FF) are significantly smaller in body size than MM:MM and FF:FF genotypes (post hoc *t*-test P value range <0.001 to <0.05; Fig. 5e) where M and F refer to the marine and freshwater alleles, respectively.

Carrying recombinant copies of chromosome IV may also reduce the probability of survival: the genotype composition of the cohort changed over time, with significantly higher minimum crossover counts among the five adaptive loci in fry (sampled July and August) compared with juveniles sampled 2–3 months later (September and October; mean crossover number in fry 1.845, juveniles 1.313; *t*_233.93_ = 3.675, *P* < 0.0003). Consistent with this, pairwise LD among adaptive loci increased as the cohort aged from fry to juveniles (Fig. 5f). In contrast to fry, older juveniles are more likely to carry ecotype-matched than mismatched alleles at the five adaptive loci—an effect plausibly explained by higher mortality of recombinant individuals over time. Combined, these results suggest that individuals carrying mismatched marine and freshwater alleles caused by recombination among adaptive loci on chromosome IV grow and survive less well than their non-recombinant peers.

## Discussion

Linking recombination variation to its evolutionary consequences in natural populations is a major challenge. By constructing individualized high-resolution one-generation crossover maps in threespine stickleback fish, our empirical study provides ecotype- and sex-specific estimates of recombination rate unconfounded by the varying effects of demography and selection in different populations. This perspective complements previous approaches that used sex-averaged population genetic estimates such as *rho*^15,45,66–68^. To investigate the molecular basis of recombination regulation, we juxtapose our sequencing-based maps of crossover locations with their molecular features including meiotic DSB hotspots. Finally, studying a natural marine–freshwater hybrid zone, we show evidence of deleterious fitness consequences of recombination in the wild.

Sex differences in recombination are abundant across taxa and among fishes, species with male and female biases are equally common^47^. We found strong, 1.76-fold female-biased heterochiasmy (Fig. 1d), consistent with previous reports^9,46^. In a male stickleback gamete, approximately half the chromosomes are inherited faithfully without a recombination crossover. While surprisingly low, this is consistent with meiotic crossover assurance requiring an obligate chiasma on each of the 1*n* = 21 homologous chromosome pairs. Non-recombinant chromosomes in gametes can thus arise when genetic exchange occurs between only two of the four sister chromatids in the obligate chiasma, and subsequent segregation into four haploid gametes gives two with recombination crossovers and two without. Assuming no transmission bias, an approximate minimum estimate of total chiasmata per meiosis is double the per meiotic product count (males = 10.81 × 2 = 21.62 chiasmata per meiosis across 21 chromosome pairs; females = 19.06 × 2 = 38.12). Since at least one crossover is necessary for stable pairing and proper segregation of chromosome homologues, this probably represents a mechanistic constraint imposing a lower limit on recombination rate. Given that stickleback males recombine close to this lower limit, evolution of further recombination suppression is probably constrained. In contrast, females transmit considerably more crossovers, are therefore probably less constrained and may lower recombination rates without segregation failure. This provides a plausible explanation as to why we detect significant recombination suppression in female but not male hybrids.

Stickleback crossovers are biased towards the subtelomeric regions of the genome (Fig. 1e), and while haploid selection against male gametes with mid-chromosome crossovers cannot be fully excluded, our previous studies of empirical recombination crossovers in male sperm show a similar subtelomeric bias^58^. This subtelomere bias is generally attributed to conserved meiotic processes, including chromosome condensation, centromere position, telomere bouquet formation and crossover interference^19,57,69,70^, the latter of which is considerably stronger in males than females (Supplementary Fig. 4). Interestingly, gene density is uniform across the length of the chromosomes (Supplementary Fig. 8) but loci underlying parallel divergent adaptation^32^ are found predominantly in recombination-reduced inner regions, suggesting the broad-scale recombination landscape shapes the genetic basis of ecotype divergence.

Linear modelling quantifying the effects of sex and ecotype on recombination rate, revealed that while sex is a major predictor, ecotype and the interaction of sex and ecotype explain recombination variation in more than 20% of the genome (Fig. 2a,b). The number of genomic regions with higher freshwater recombination rate than marine is similar to the number with higher marine recombination rate than freshwater (Fig. 2b). Comparing local recombination rates between parental ecotypes and F1-hybrid offspring reveals the ecotype effect is partially recessive (Fig. 2e). Importantly, F1 hybrids tend to recapitulate the recombination rate of the lower recombining parent independent of that parents’ ecotype or sex. If this pattern of recombination variation has a molecular basis, we interpret this as evidence for a local genomic modifier (for example, *cis*-acting) whose effects manifest in heterozygotes/hybrids. We argue that it is unlikely to be caused by divergence in a *trans*-acting recombination modifier, since *trans*-acting effects would be expected to proceed in the same direction across the genome (for example, a non-synonymous mutation in one ecotype causing more efficient DSB initiation (PRDM9 or Spo11) or repair (for example, RAD51) would be expected to increase recombination at all sites across the genome. Rather, a more parsimonious mechanism explaining the observed effects would be changes in local *cis*-acting genomic modifiers (local genomic DNA sequence). For example, a mutation in a local genomic region in one ecotype may interfere with local crossover formation either through disruption of DNA recognition motifs used by recombination machinery^60^ or interference of crossover repair due to DNA mismatches (heterozygosity) in homologous templates^59^. Accordingly, heterozygosity itself may manifest as a local *cis*-acting effect in F1 hybrids by interfering with recruitment of machinery to the site or impeding homologous template repair, or both.

This raises the question of how precisely recombination location is controlled in sticklebacks. Organisms lacking PRDM9 are thought to represent the ancestral state of recombination regulation, in which DSBs occur preferentially in accessible chromatin, leading to a conserved pattern in crossover distribution^44,71,72^. Despite lacking PRDM9, stickleback crossover distribution is non-random and more similar to species with intense PRDM9-mediated hotspots (Fig. 3a). These ‘semi-hot’ hotspots follow the genomic pattern of DSB hotspots determined from DMC1 ChIPseq (Fig. 3b,c) and provides strong evidence for crossover frequency being regulated by factors influencing DSB initiation. Additionally, the distinct class of crossovers far away from promoters showing reduced chromatin accessibility imply as yet unknown mechanisms, other than PRDM9, in direct DSB formation (Fig. 3e).

Variance in recombination introduced via heterochiasmy and recombination suppression in heterozygotes may have evolutionary implications for populations undergoing adaptive divergence with ongoing gene flow. Adaptive alleles are shuffled at an exceptionally low rate in hybrid females and hardly at all in hybrid males, and the recombination rate is lower in local genomic regions of high heterozygosity (Fig. 4a–c). In a natural marine–freshwater hybrid zone, evidence suggests allelic shuffling among five loci underlying adaptive divergence has deleterious fitness effects including reduced growth and survival (Fig. 5). We hypothesize this may form the basis by which natural selection has favoured recombination modifiers that suppress recombination in hybrids and heterozygotes.

Our study design precludes us from attributing causality between ecotypic divergence and recombination suppression. It is plausible that individual adaptive loci offer fitness benefits to individuals (for example, physiological, morphological or behavioural advantages) independent of any resulting divergence, heterozygosity and recombination suppression that may later evolve as a consequence of divergent selection. Regardless, our population genetic simulations show that *cis*(coupled) recombination suppression due to heterozygosity can increase the strength and maintenance reproductive isolation (barriers to gene flow) between adaptively diverging ecotypes. Simulations reveal that when new adaptive alleles arrive in linkage with other beneficial mutations, heterochiasmy helps maintain greater divergence between marine and freshwater populations (Supplementary Figs. 9 and 11, Supplementary Table S8), while recombination suppression in cis(coupled)-heterozygotes increases the rate at which new beneficial mutations establish and spread through populations maintained in selection–migration balance (Supplementary Table 8). Combined, our results highlight how both molecular constraints and population-level evolutionary forces have the potential to shape the recombination landscape.

Leveraging multiple study approaches on stickleback fish populations undergoing adaptive divergence with ongoing gene flow, we quantified fine-scale variation in recombination across the genome, showed evidence and possible molecular mechanisms for recombination suppression in hybrids and the fitness consequences of recombination among loci underlying divergent adaptation in the wild. Our results support predictions that divergence in *cis*-acting recombination modifiers whose mechanisms are disrupted at local genomic regions of heterozygosity and in hybrids across the entire genome may have an important role to play in the maintenance of differences among adaptively diverging populations. Further studies are needed to address the role of the strong sex bias in recombination rate and to identify the molecular mechanisms through which natural selection shapes recombination.

## Methods

### Generating data for individualized crossover maps

Stickleback fish were collected from the River Tyne, East Lothian, Scotland. Marine and freshwater sticklebacks were caught from 4 km and 19 km, respectively, from the river mouth during May–June 2014. Following the standard protocol, in vitro fertilization crosses were carried out in the field and the embryos were raised in a stickleback fish facility at the Max Planck campus in Tübingen, Germany. Both marine and freshwater fish were raised in 10% seawater salinity (3.5 ppt) with daily 10% water changes. All fishes were fed once a day with the same food that consists of both marine and freshwater invertebrate diets. For fine-scale individualized recombination map construction, 18 nuclear families consisting of 90–94 offspring per family were generated by in vitro fertilization of 36 distinct parent individuals (six marine ♀ × marine ♂ crosses, six freshwater ♀ × freshwater ♂ crosses, three (freshwater × marine) ♀ × (freshwater × marine) ♂ crosses and three (marine × freshwater) ♀ × (marine × freshwater) ♂ crosses). Of these 18 crosses, two freshwater and two marine crosses were carried out in the field (family×1, family×4, family×11 and family×20) whereas 14 crosses were made in the laboratory using offspring raised in aquariums. For crosses that resulted in a small clutch size (<90 eggs), a second round of in vitro fertilization was carried out the following breeding season with eggs from the same female and cryo-preserved sperm from the same male fish. DNA was extracted either from a piece of tail fin (parents) or from the whole fish body (offspring at age of 1 month) following the solid-phase reverse immobilization bead-based protocol^73^. Multiplexed DNA extraction was carried out using a TECAN liquid handling robot. A custom low-cost, high-throughput library preparation protocol, adapted from ref. ^74^ was followed (detailed in Supplementary Methods 2). Libraries (insert size ~250 bp) were 150-bp paired-end sequenced on an Illumina HiSeq3000 sequencer to approximately 10× genome coverage per offspring in each family and about 60× coverage per parent (Supplementary Table 1). Sequenced reads were mapped to the stickleback reference genome gasAcu1 (Broad S1 assembly generated from an Alaskan freshwater female)^32^ using Burrows–Wheeler Aligner v0.7.10-r789 (ref. ^75^) with ‘bwa mem’. Mapped reads were then sorted and indexed using SAMtools^76^. Further read processing until variant calling was carried out according to the best practices recommended by Genome Analysis Toolkit (GATK)^77,78^ (described below).

### Variant calling and filtering

Individual variant calling was carried out using HaplotypeCaller in GATK v3.4. Individual gVCF files from a family were then combined using GenotypeGVCF in GATK v.3.7. This step creates a joint genotype file for a family with information of parents and all offspring at each variant position. High-quality variants for further analysis were then selected based on the following criteria: (1) SNPs (indels are excluded), (2) biallelic, (3) heterozygous in either parent, (4) SNPs with quality score greater than first quantile of the quality score distribution of the whole family dataset, (5) model-based clustering analysis of allele frequency was carried out using R package mclust version 5.4.1 (ref. ^79^) since in each family, alleles segregate in Mendelian ratio, SNPs falling in tight clusters of allele frequency around 0.25, 0.5 and 0.75 were selected, (6) SNP filtering based on variant criteria (QD (Variant confidence/Quality by depth) <2.0 || FS (Phred-scaled *p*-value using Fisher’s exact test to detect strand bias) >60.0 || MQ (Root mean square of the mapping quality) <40.0 || MQRankSum (Z-score From Wilcoxon rank sum test of alternate versus reference read mapping qualities) <−12.5 || ReasPosRankSum (Z-score from Wilcoxon rank sum test of alternate versus reference read position bias) <−8.0) and (7) SNPs with coverage not differing by more than 50% of mean coverage and read bias between alleles less than 30% (Supplementary Table 1).

### Scaffold orientation correction

The stickleback reference genome, Broad S1 assembly (gasAcu1) was used for the initial read placement. However, misorientation of 13 scaffolds in this original assembly has since been rectified^80^. The order of SNPs in these 13 scaffolds was reversed before haplotype phasing using a custom Perl script. It should be noted that only the orientation of scaffolds in the assembled part of the genome was corrected. In contrast to the latest version of the assembly, no new scaffolds were additionally tied into the assembled chromosomes from unassembled scaffolds, making our crossover counts a conservative underestimate.

### Haplotype phasing

The phasing algorithm SHAPEIT^81^ in combination with duoHMM^82^ was used to phase SNPs within a family. The joint variant call file for a family was split into two files of paternally informative and maternally informative SNPs (those that are heterozygous in the father while being homozygous in the mother and vice versa; SNP counts in Supplementary Table 1). SHAPEIT and duoHMM require a genetic map for each chromosome as an input. To avoid any bias in phasing due to the pre-existing recombination rate per window, a linear genetic map with a 3 cM Mb^−1^ (reported genome-wide average recombination rate in sticklebacks is 3.11 cM Mb^−1^ (ref. ^42^)) recombination rate was used. The first round of phasing was then carried out with SHAPEIT without pedigree information. The SHAPEIT output files were then used in duoHMM to identify and correct phasing errors by taking pedigree structure into consideration. In addition, duoHMM generates a list of SNPs with genotyping error probability. After one round of SHAPEIT and duoHMM, problematic SNPs were shortlisted based on the two following criteria;

1. SNPs with genotyping error probability >0.9 in 20 or more offspring
2. SNPs showing biased transmission distortion (phased to one haplotype in more than 75% of offspring). While some transmission bias may be biologically real due to processes such as meiotic drive, it may also be bioinformatic error. These SNPs were therefore removed and a second round of phasing (SHAPEIT and duoHMM) was then performed.

### Crossover identification

We developed an R-based pipeline employing the following strategy to identify crossover events: long switches (haplotype switches with >50 kb size on either side and 50 or more SNPs supporting each haplotype) in phased haplotypes were identified as true crossover events, filtering out all small switches that are either genotyping errors or gene conversion events. After calling crossover events from every sequenced offspring, further filters were applied to the crossover list to remove false positive events detected as a result of phasing error or low sequencing coverage: (1) crossovers appearing in 50% or more offspring in a family between the same boundary SNPs (probably caused by parental phasing error) were removed, (2) all crossovers from offspring with sequencing coverage <2× were removed, (3) crossovers with low resolution (>1 Mb) were removed unless due to the lack of informative SNPs and (4) crossovers at inversion boundaries were removed (further details of this filter can be seen in the section on inversions).

### Linear modelling of sex and ecotype variation

We performed linear modelling on 500 kb windows across the genome using individual recombination rate as the response variable and sex, ecotype or an interaction term with both, as explanatory variables using the ‘lm’ function in R. Only windows with non-zero recombination rate in at least one individual were included in the analysis. A minimal model explaining variation in recombination rate was obtained by applying the ‘step’ function. The genomic location of crossovers was shuffled 100,000 times to estimate the FDR of detecting an effect in each window. Windows with FDR <0.05 were shortlisted. In a window where sex and ecotype explain significant variation, effect size was calculated by subtracting mean estimates.

### Additivity/dominance in F1 hybrids

In windows with a significant ecotype effect, deviation in hybrid recombination rate from mid-parental recombination rate (mean recombination rate of parental ecotypes in the respective windows) was calculated as hybrid mean minus mid-parental mean. A value of zero indicates additivity in hybrids whereas a positive value indicates dominance of high recombining allele and a negative value indicates dominance of low recombining allele in hybrids. This analysis was performed at three different scales (5 kb, 50 kb and 500 kb) and in both a sex-independent and sex-dependent manner.

### Genomic ancestry calculation from whole-genome sequencing data

We estimated the probability of marine, freshwater or hybrid ancestry at each SNP position in each parental individual based on allele frequency estimated from 11 freshwater and 10 marine fish sequenced in the Jones et al.^32^ study. In the 21 genome dataset, a genotype observed at high frequency in marine individuals was defined as a marine genotype and the freshwater genotype was defined similarly. The probability of ancestry at each site in each parental genome of this study is assigned based on the number of reads covering the marine or freshwater allele. Mean ancestry probability in regions of interest such as adaptive loci and adaptive islands were then calculated and further correlated with recombination rate to investigate the effect of regional ancestral allele state on recombination using a non-linear quadratic regression:

Recombination Rate ~ β_1_genomic_ancestry^2^ + β_2_genomic_ancestry + Sex + ε(error)with significance of the quadratic term tested using an ANOVA *F*-ratio test.

### Recombination at adaptive loci

For comparison of recombination rate within and immediately flanking adaptive loci, we first rescaled 242 adaptive loci^32^ to a fixed width and calculated the mean and standard deviation of recombination rate in sliding windows across the adaptive loci and flanking regions. Adaptive loci were further grouped based on mean local genomic ancestry and the analysis repeated.

### Identifying candidate hotspots that differ among ecotypes

The 1-bp midpoint of crossover intervals from each individual were pooled by ecotype and down-sampled to create three equal sized sets of 9,463 marine, freshwater and hybrid crossovers (excluding crossovers spanning scaffold boundaries and/or those with interval boundaries exceeding 10 kb). The number of crossovers within 5 kb of each midpoint was counted for all three ecotypes, and the log_2_-fold change ratio of crossover counts was calculated for pairwise ecotype comparisons using a pseudocount of 0.1. The significance of the fold change in crossover count being different from zero was determined by comparison to the upper and lower 0.025 tails of fold change values from a null distribution, accounting for mean average crossover count (similar to MA analyses of log ratio (M) versus mean average (A) in analysis of differential gene expression). Specifically, this null distribution was created for each pairwise ecotype comparison by shuffling the down-sampled ecotype crossover locations into two random sets of 9,463 crossovers 10,000 times, counting the number of crossovers in each set in 5 kb windows and calculating the log_2_-fold change ratio of crossover counts in pairwise comparisons among sets. The 2.5% quantiles of the fold change distribution for a given mean average number of crossovers were then calculated and used to identify observed fold change ratios that would occur by chance with a probability of less than 5%. This set of 5-kb windows with significant fold change in crossover counts was further filtered to consider only those with six of more crossovers among the fish being compared. Assuming a uniform distribution, the probability of this happening by chance (6 of 18,926 crossovers falling within 5 kb of each other) is 1.6 × 10^−30^. Neighbouring or overlapping windows were then merged to describe genomic regions containing candidate ecotype-divergent hotpots.

### Inversions

Structural rearrangements such as inversions are common in natural populations and may segregate within the nuclear families used in this study. Parents may be heterozygous or homozygous for DNA sequences that show opposite orientation to the reference genome assembly. In regions where the genomic orientation of the sample is inverted compared with the reference genome and there is a crossover within that region, there will be false positive crossovers called at both inversion boundaries. Such events will leave a signature distribution of triplet crossovers within a short physical distance. The list of crossovers was examined to find such triplets where the first and third crossover occurred within 2 Mb physical distance. Across the genome, eight regions with multiple offspring showing inversion triplets in the same genomic location were detected on seven different chromosomes (Supplementary Table 7). For these triplets, the first and third crossovers were removed and the position of the second crossover was corrected according to the inverted orientation.

### Investigating fitness effects of recombination in a natural hybrid zone

We leveraged the power of a previously described natural marine–freshwater stickleback hybrid zone with large population sizes to explore the potential fitness effects of recombination in the wild^65^. DNA from 1,045 fish fin clipped from eight sites along the River Tyne, East Lothian Scotland^65^, was genotyped at 3,084 SNPs across the genome using an Illumina Golden Gate custom genotyping array. Genotypes were called using Illumina’s GenomeStudio (Genotyping module) software (RRID: SCR_010973), with manual clustering of genotype classes at each of the 3,084 loci. After removing those lacking three clearly distinct genotype clusters (AA, AB and BB), or with excessive failure rates across individuals (≥20%), 2,265 variants were retained for further analyses. Individuals with missing genotypes at ≥20% of markers were removed. Genome-wide genetic ancestry analysis (calculated following methods described in Jones et al.^65^) shows that populations from rockpools (site 1) and freshwater locations (sites 5–8) at opposite ends of the hybrid zone have pure marine and freshwater genetic ancestry, respectively, while populations in the lower reaches of the river (sites 2–4) comprise marine, freshwater, hybrid and recombinant individuals. Adult sticklebacks in the River Tyne die shortly after breeding in the summer, resulting in almost completely non-overlapping generations. This provides a valuable opportunity to study cohorts through time, including the differential growth and survival of pure and admixed genotypes.

Fry and juveniles from a single breeding year cohort were sampled from the centre of the hybrid zone (sampling sites 2, 3 and 4, as described in Jones et al.^65^) on a periodic basis (approximately every 4–5 weeks from June through to December 2003. Young-of-the-year comprising 103 fry (<30 mm in size) and 182 juveniles (30–50 mm) were trapped using wire mesh minnow traps (5 mm mesh size), fin clipped, tagged with an elastomer dye and released, and their DNA genotyped as above. Recaptured individuals (approximately 10% of a catch) were released immediately without fin clipping, ensuring no individual was genotyped twice. We therefore assume these recaptured individuals represent a random sample of genotypes from the population.

We explored the fitness effects of recombination in the wild in two ways: using standard length as a proxy for fitness (ability to grow) and changes in within-chromosome IV two-locus LD over temporal samples as an indication of differential genotypic survival. All analyses were performed in R^83^.

#### Association between crossovers and residual standard length

Residual standard length reflecting the relative size of the individuals compared with the mean of their sampling date, was calculated for the young-of-the-year sampled from the centre of the hybrid zone by removing the effect of sampling date as a factor (residuals of a linear regression):


resid(lm(standard_length ~ as.factor(date)))


Stickleback chromosome IV includes five genomic regions with extraordinarily strong molecular signals of parallel marine–freshwater divergence (Fig. 3a; *eda* chrIV:12.81, *Stc2* chrIV:13.94, *pnpla3* chrIV:22.21, *pparaa* chrIV:26.30 and CDPF1 chrIV:26.35)^32^. Focusing on chromosome IV, and using SNPs within these regions, we calculated the minimal number of crossovers needed to explain an individual’s multi-locus genotype at the five above-mentioned adaptive loci. Importantly, this conservative (underestimate) count of crossovers is distinct from a ‘hybrid index’ that is commonly used in hybrid zone studies. Here, individuals carrying a large number of mismatched marine and freshwater genotypes at the five loci will have a high score (maximum possible score for the five loci studied is eight) while marine, freshwater and F1 hybrids (heterozygous for non-recombined marine and freshwater chromosomes) will have a low score (minimum possible score is 0).

We then used linear modelling to ask whether significant variation in residual standard length can be explained by the number of crossovers among chrIV loci as an independent variable:


lm(residual_standard_length ~ minimum_Crossover_Number)


We next tested whether the negative relationship between standard length and minimum crossover number can be explained by genotypes at individual adaptive loci by adding an interaction term, for example:


lm(residual_standard_length ~ minimum_Crossover_Number * chrIV_12812500)


Given the non-significance of interactions in all models we minimized the models to test for significance of genotype as a main effect (all *P* values >0.05). For example:


lm(residual_standard_length ~ minimum_Crossover_Number + chrIV_12812500)


Finally, we found an individual’s residual standard length could not be explained by genotype at any of the five adaptive loci alone. For example,


lm(residual_standard_length ~ chrIV_12812500)


Next, we asked whether variation in residual standard length can be explained by pairwise multi-locus genotypes at two of the five adaptive loci. For example,

lm(residual_standard_length ~ chrIV_12812500 * chrIV_23968000) and found significant interactions between *eda:Stc2*, *eda:pnpla3*, *Stc2:pnpla3*, *Stc2:CDPF1* and *Stc2:pparaa*. We extracted the estimated coefficients and their standard errors from these models and on post hoc two-sided *t*-tests found recombinants (individuals with mismatched genotypes at the two loci) to be smaller in size than individuals of pure genotype.

#### Within-cohort changes in LD on chromosome IV

We selected a subset of 31 variants spanning chromosome IV with minor allele frequencies ≥0.3 within the young-of-the-year sampled from the hybrid zone (four variants tagging each of the five above-mentioned adaptive loci and an additional 11 variants spanning neutral regions along chromosome IV). Using the ‘cALD’ function within R library ‘pould’^84^ we calculated LD statistics *R*^2^ from unphased genotype data for ‘fry’ (sampling dates June–July), and ‘juveniles’ from the same cohort sampled later in the year (sampling dates September–December). While it is unlikely given the low recapture rate, we cannot rule out the possibility that recaptured individuals were specifically enriched for genotypes of mixed marine and freshwater alleles across adaptive loci on chromosome IV and thus their absence in the juvenile cohort contribute towards the observed changes in multi-locus genotypes and LD between fry and juveniles.

### DMC1 antibody production and validation for ChIPseq

To produce antibodies against stickleback DMC1 protein, we expressed stickleback DMC1 as a codon-optimized (GeneArt, Thermo Fisher Scientific) recombinant protein (vector pColdTM TF-DNA, Takara Clontech) for use as the antigen. Following transformation into the expression strain BL21 DE3, a single colony was inoculated in 20 ml Lysogeny broth broth containing 100 μg ml^−1^ ampicillin for overnight incubation. Then, 10 ml of the overnight culture was inoculated into 500 ml Lysogeny broth without antibiotic grown to OD 0.4–0.9. Once the culture acquired optimal concentration, it was incubated on ice for 30 min and 0.5 mM isopropyl-β-d-thiogalactoside was added. Protein expression was carried out overnight at 15 °C and then purified using Ni-NTA columns. The purified recombinant protein was used as an antigen in an 87-day immunization programme on guinea pigs (performed by Eurogentec). The DMC1 antibody was then purified from antisera following affinity purification (using Affi-Gel 15 from Bio-Rad) protocol. Western blot, ChIP and immunohistochemical staining of histological sections on testes material were used to verify sufficient specificity and affinity (Supplementary Fig. 17).

### ChIPseq

Sticklebacks are seasonal breeders and during early winter the testes are enriched for primary spermatocytes undergoing the first round of meiosis^85^. In the fish facility, the fish are reared in a cycle of 3 months long day length (16:8 h light:dark) and 3 months short day length (8:16 h light:dark). Using hematoxylin–eosin staining on histological sections of testes, we determined that in our fish facility, the proportion of primary spermatocytes was maximal (approximately 30%) during the last 3 weeks of the 3-month short day length period (Supplementary Fig. 17). Testes and livers were collected during this time window, snap-frozen in liquid nitrogen and stored at −80 °C.

For ChIPseq of proteins of interest, we used a pool of about 20 testes, and 20 pieces of liver tissue as a negative control. A sequential pull down with homemade DMC1 antibody followed by commercial H3K4me3 antibody (Millipore, 07-473) was carried out with the same tissue lysate. DMC1 ChIP and library preparation were carried out following the protocol described in refs. ^55,86^. The kinetic enrichment of single-stranded DNA was excluded for H3K4me3 ChIP. The libraries were 150-bp paired-end sequenced on an Illumina Hiseq 3000. After sequencing, DMC1 ChIP reads were trimmed to 40 bp using Trimmomatic^87^, and a specialized bioinformatic pipeline described in Khil et al.^55^ was used for processing the reads. Peaks in the DMC1 ChIP data in comparison with input were called using MACS2 (v2.1.1)^88^ with the following parameters: -q 0.1 --nomodel --slocal 5000 --llocal798 10000 --extsize 800 -f BED --SPMR -g 463000000 -B. For H3K4me3 ChIP reads, macs2 peak calling was done using default parameters.

### ATACseq of primary spermatocytes

Using hematoxylin–eosin staining on histological sections of testes^85^, we determined that in our fish facility, the first round of meiosis in males occurred during the last 3 weeks of the 3-month short day length period. Testes were dissected from adult males at this time for the assay for transposase-accessible chromatin sequencing (ATACseq) and processed immediately after dissection. The isolation of primary spermatocytes by flow cytometry assisted sorting was carried out using a BD FACSMelody Cell Sorter (BD Biosciences): initially, 4,6-diamidino-2-phenylindole staining for chromatin content (4C primary spermatocytes, 2C secondary spermatocytes and 1C spermatids/sperm) and microscopy were used to optimize the gating strategy that was eventually based on FSC-A and SSC-A alone (~cell size and complexity) since these sufficiently differentiated between the primary spermatocytes, secondary spermatocytes and spermatids/sperm (Supplementary Fig. 18). Briefly, for each ATACseq^89^ replicate, the testes of three males were pooled, dissociated with 0.07 U ml^−1^ liberase (Sigma) and 50,000 primary spermatocytes were isolated using flow cytometry. Cell membranes were lysed with cold 10 mM Tris pH 8, 10 mM NaCl, 3 mM MgCl_2_ and 0.1% NP-40, and nuclei were isolated by centrifugation. Tn5 was applied onto the nuclei in standard TAPS-DMF (N-[tris(hydroxymethyl)methylamino]propanesulfonic acid-Dimethylformamide) buffer for 30 min at 37 °C with shaking (1,000 r.p.m.), the tagged DNA was cleaned with the Min-Elute kit (Qiagen) and processed to a sequencing library. A control library of naked genomic DNA was also made from each testes pool. The libraries were sequenced on an Illumina platform, the reads mapped to GasAcu1, mitochondrial reads and PCR duplicates were removed, the files were down-sampled to the level of the limiting coverage (20M read pairs) and MACS2 was used to call peaks (--shift -100 --extsize 200 --tsize 150). Three marine replicates and three freshwater replicates were analysed. The replicates of both ecotypes were pooled for comparison to crossover coordinates and anti-DMC1 ChIPseq peaks.

### Reporting summary

Further information on research design is available in the Nature Portfolio Reporting Summary linked to this article.

### Supplementary information

Supplementary Information Supplementary Figs. 1–18, Tables 1–9 and Methods 1 and 2.

Reporting Summary

## Data Availability

The datasets generated and analysed during the current study are available in the National Center for Biotechnology Short Read Archive and Gene Expression Omnibus data repositories under BioProject Accession PRJNA1062151 and GSE254557 (https://www.ncbi.nlm.nih.gov/geo/query/acc.cgi?acc=GSE254557; then enter token 'ajehoskedxobzmn') are publicly available on publication.

## Code availability

Source code for population genetic simulations performed in this manuscript can be found at GitHub (https://github.com/felicitycjones/Venu_SticklebackRecombination)^90^.
