# Supplementary material for: Fine-scale contemporary recombination variation and its fitness consequences in adaptively diverging stickleback fish
Source: Nat Ecol Evol. 2024 Jun 5;8(7):1337–52. doi: 10.1038/s41559-024-02434-4 (PMC11239493; doi:10.1038/s41559-024-02434-4)
Supplement: Supplementary file 1 — Supplementary Figs. 1–18, Tables 1–9 and Methods 1 and 2. [file 41559_2024_2434_MOESM1_ESM.pdf]

nature ecology & evolution

Supplementary information

https://doi.org/10.1038/s41559-024-02434-4

Fine-scale contemporary recombination variation and its fitness consequences in adaptively diverging stickleback fish

In the format provided by the authors and unedited

Supplementary material

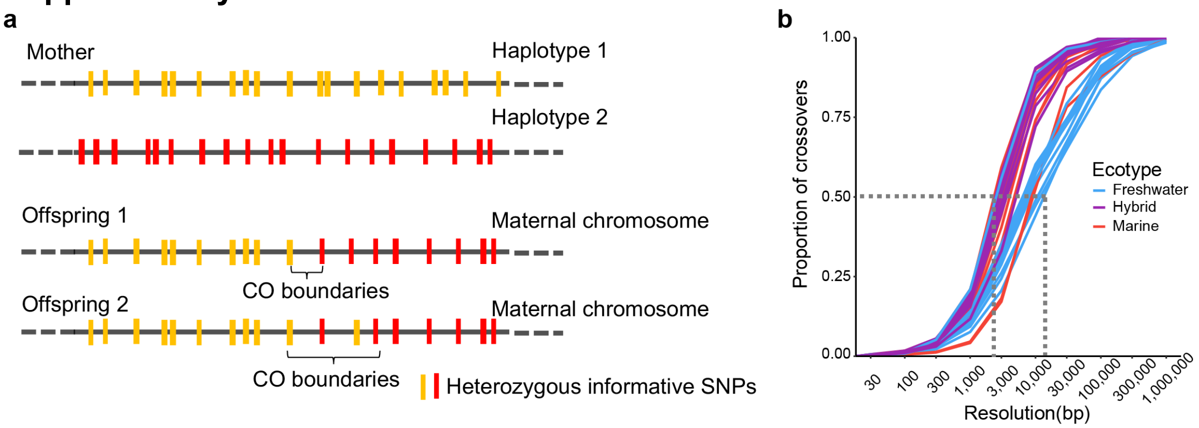

Supplementary Figure 1: Crossover definition: *a) From phased chromosomes of a parent and offspring, a crossover is defined as an interval flanked by the last SNP of the first haplotype and the first SNP of the second haplotype (eg: offspring 1). In a scenario such as in offspring 2, where two or more switches occurred within the minimum required block size (50kb) of each other, the crossover location was defined by the last SNP of the first large phase block and first SNP of the next large phase block. b) Fraction of crossovers in parents with their respective resolution (interval size within which crossover is identified) is shown. Median crossover resolution is marked with dotted lines.*

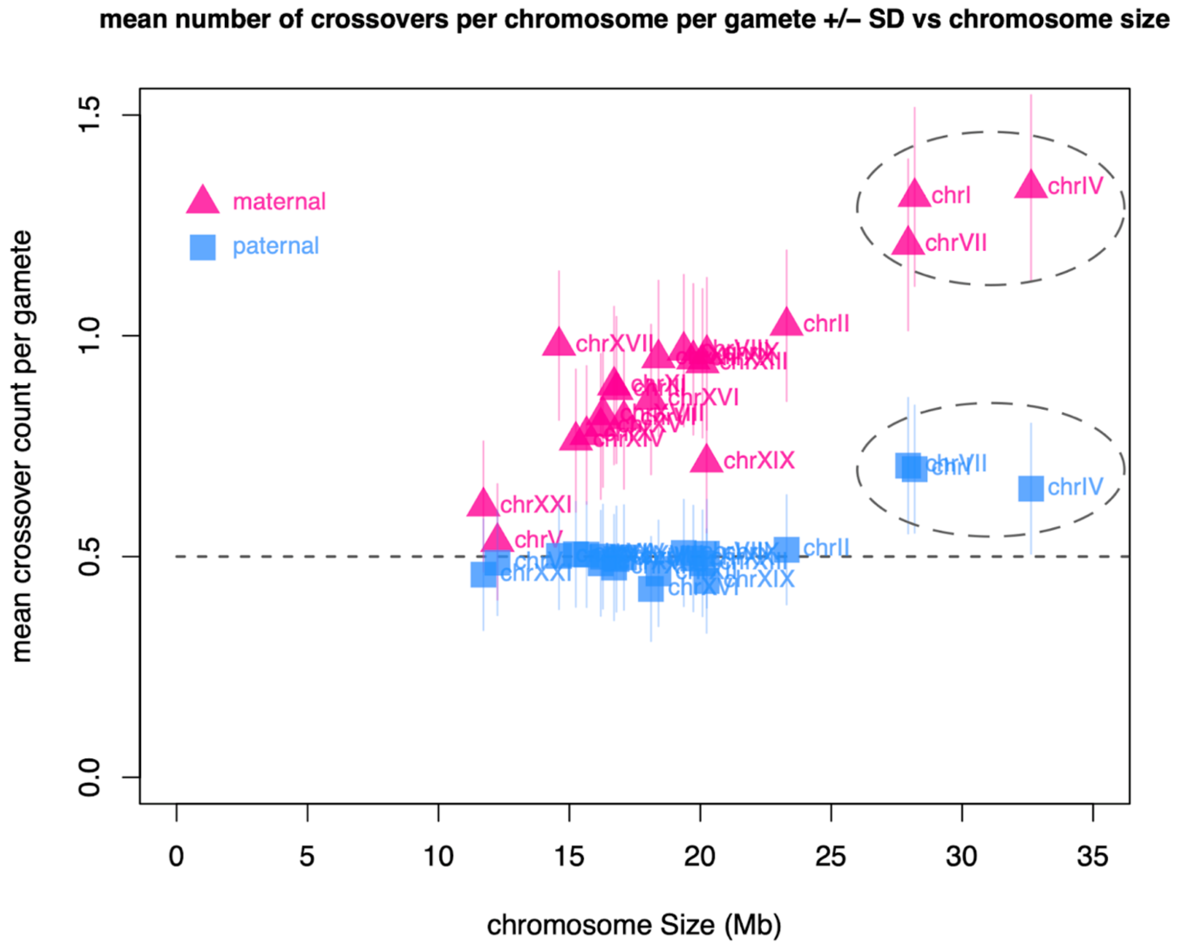

Supplementary Figure 2: The number of maternal, but not paternal, crossovers per chromosome increases with chromosome size and is highest in large chromosomes that evolved via fusion of ancestral chromosomes. *Points represent the mean number of maternal (pink triangles) and paternal (blue squares) crossovers observed on a given chromosome among offspring of all parents studied. Error bars depict the standard error among 18 male and 18 female parents studied. A strong positive relationship is observed in maternal crossovers, but less so for paternal crossovers. Dashed horizontal line at 0.5 represents the expected number of crossovers to be detected under a scenario of one obligate chiasma per homologous chromosome pair with the assumption of no bias in the transmission of resulting meiotic products to offspring. Three chromosomes larger than 25Mb (chrI, IV, and VII, shown in ellipses) have more than one maternal crossover on average, and in paternal crossovers these same three chromosomes show elevated crossover count that deviates strongly from the paternal trend of crossovers resulting from one obligate chiasma per homologous chromosome pair shown by other chromosomes.*

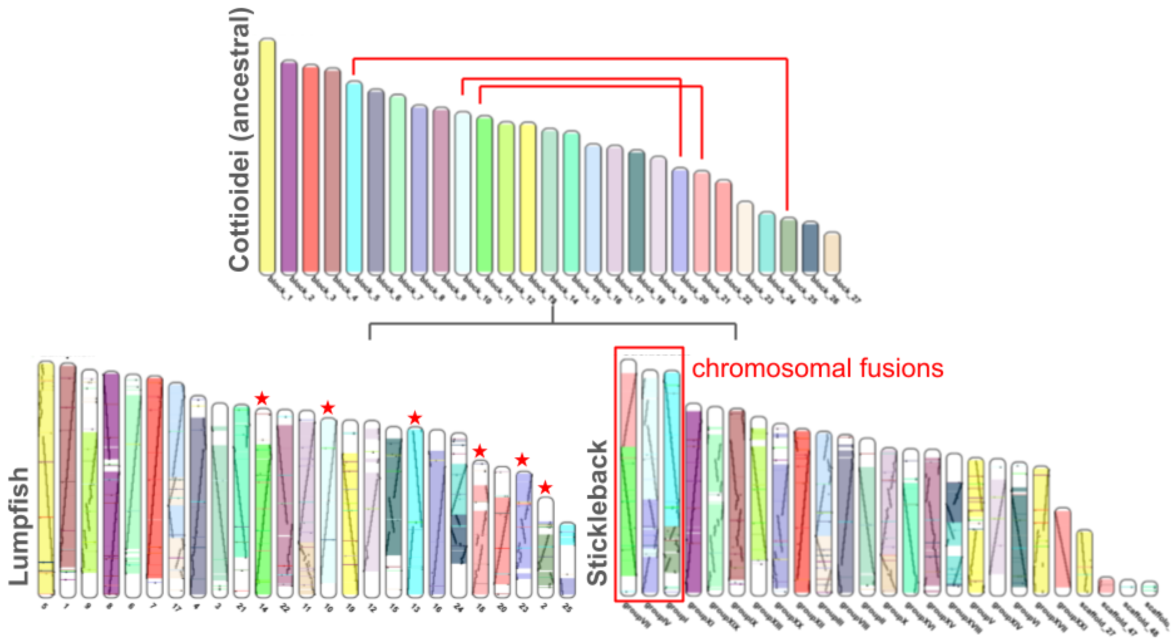

Supplementary Figure 3: Gene synteny relationships from Nguyen et al., 2022[1] among sticklebacks, lumpfish and their shared Cottioidei ancestor reveal stickleback chromosomes I, IV and VII (those with high rates of double maternal crossovers, and atypical higher rates of paternal crossovers) evolved via fusion of ancestral chromosomes. *Colors of chromosomes indicate synteny with similarly colored ancestral chromosomes, and red lines indicate fusion events in the ancestor. Present day lumpfish lack these fusions (red stars) suggesting the fusion events occurred at most within the last 100My (and most likely within the last 40Mya (chrIV), and 13-26Mya (chrVII) based on data presented in Urton et al., 2011[2] and Varadharajan et al.,2019[3] that make pairwise comparisons between threespine and fourspine, and threespine and ninespine sticklebacks respectively).*

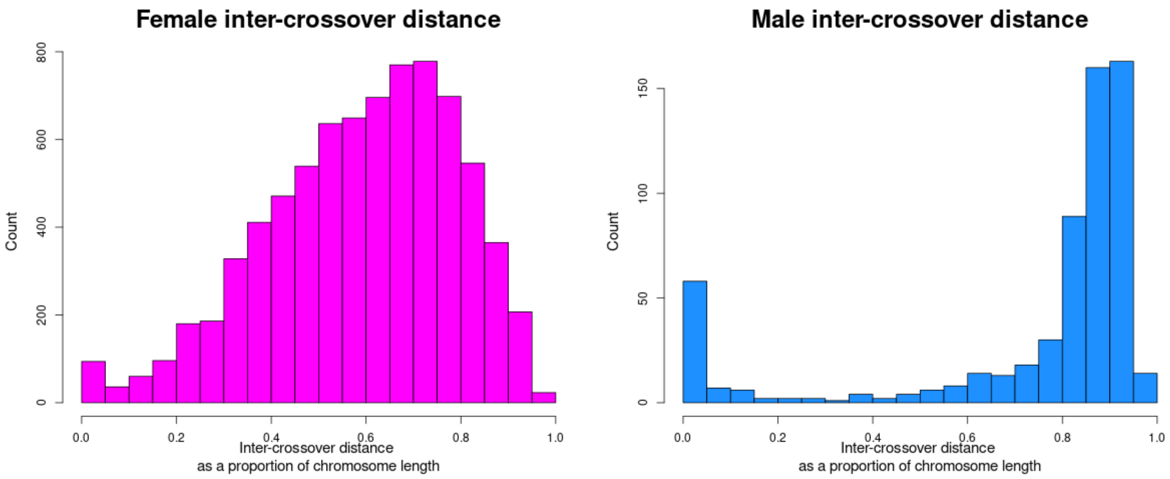

Supplementary Figure 4: Inter-crossover distance shows evidence supporting stronger crossover interference in males than in females. *Plots show the distribution of inter-crossover distances expressed as a proportion of chromosome length in females (left, N=7769) and males (right, N=603) for all chromosomes. The median inter-crossover distance is 12Mb in females and 24Mb in males. Only chromosomes with two or more maternal or paternal crossovers detected in a given offspring were considered. For each pair of crossovers being considered, inter-crossover distance was calculated as the physical distance between the midpoints of the defined crossover bed intervals (locations).*

a

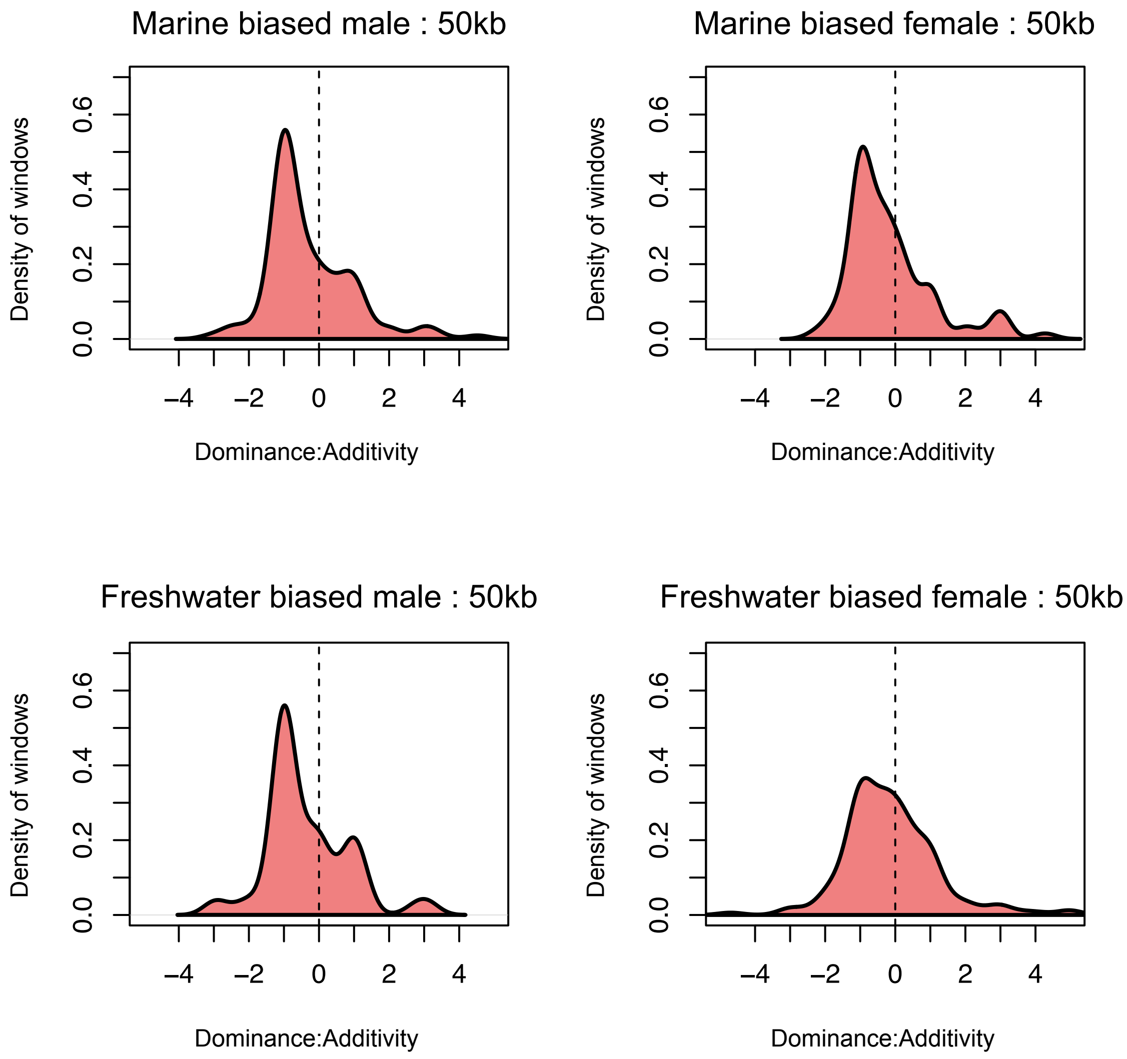

Supplementary Figure 5: Partially recessive recombination rates (partial or full dominance of low recombining parental ecotype) in both sexes of hybrids in ecotype diverging windows. *Density estimates of dominance-additivity ratio of parental ecotype crossover count in hybrid males and females in ecotype diverging windows. A value of zero indicates recombination behaves additively in F1s relative to parents, 1 dominantly, -1 recessively, and values >1 or <-1 imply over or underdominance of recombination respectively. A bias towards low recombining parental ecotype is observed irrespective of which ecotype is paternal and which maternal. Results from analysis at 50k bresolution is shown. Similar results are obtained also at 500kb and 5kb resolutions.*

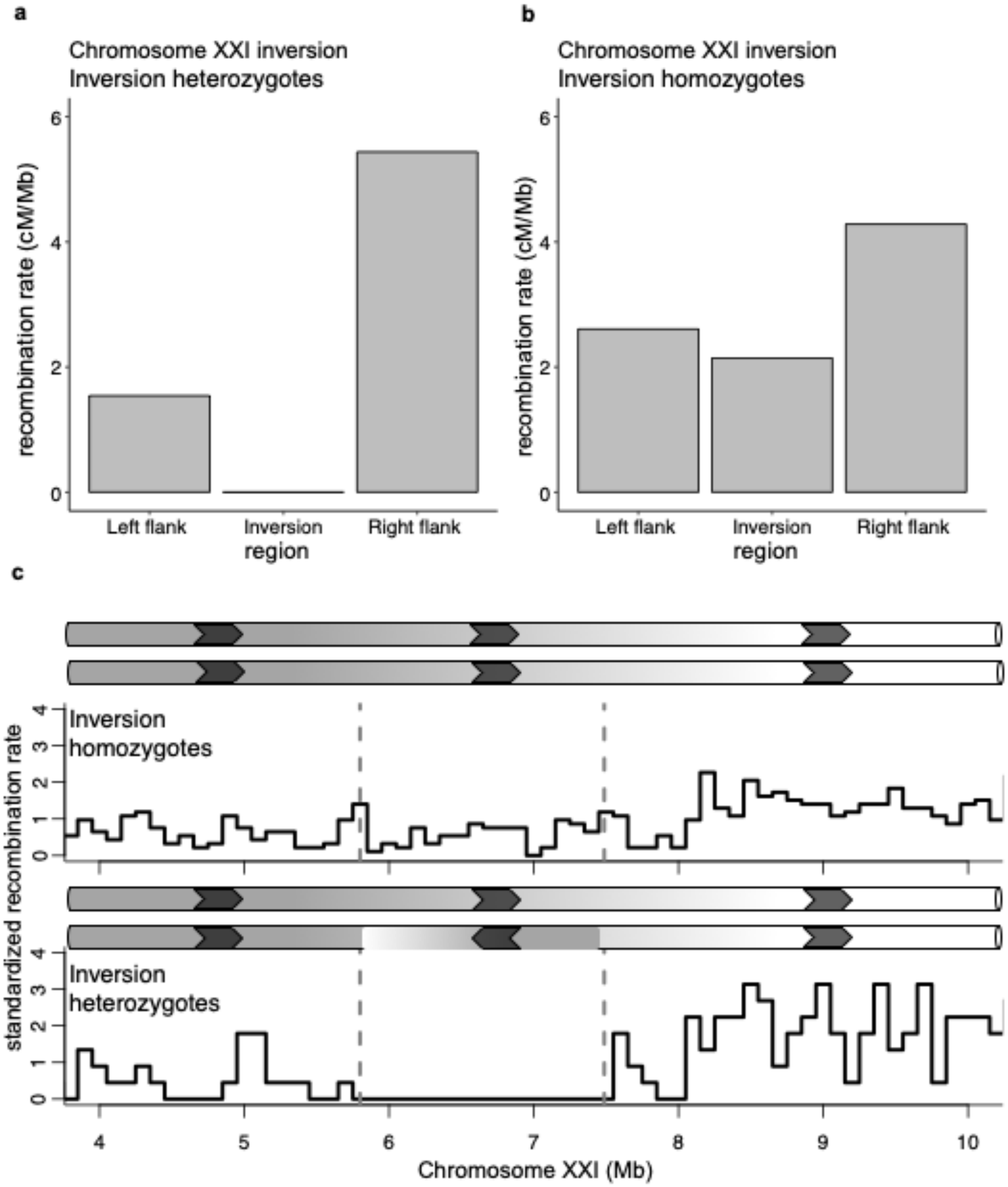

Supplementary Figure 6: Crossover suppression within chrXXI inversion heterozygotes. *Zero recombination rate within inversion in contrast to left and right flanking regions of the same size is observed in a) heterozygotes (N=25) but not in b) homozygotes (N=11). c)Standardized recombination rate (SRR) in 100 kb sliding windows across a 6 Mb region including the chrXXI inversion. Dotted grey vertical lines mark the inversion boundaries. SRR for inversion homozygotes (top panel) and heterozygotes (bottom panel) are shown with a schematic of the sequence orientation. A complete suppression of recombination within the inversion boundaries is seen in heterozygotes with compensatory increase in recombination on the right flank (closer to telomere).*

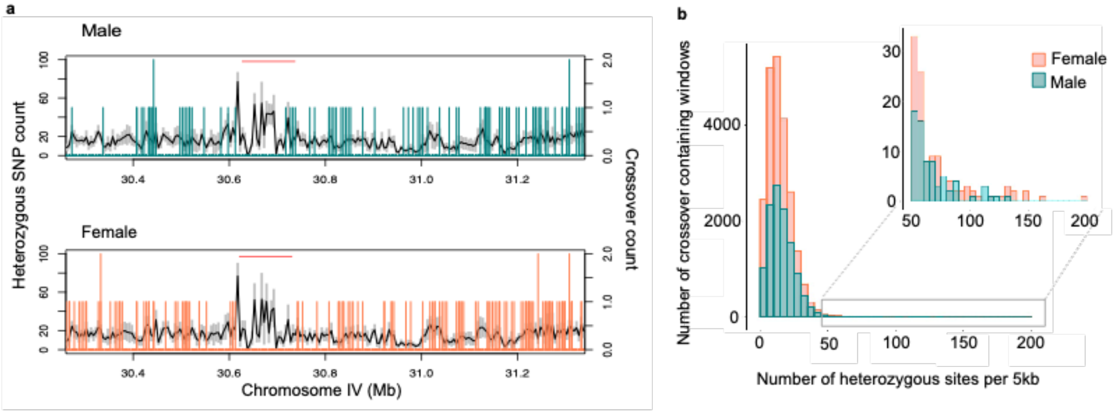

Supplementary Figure 7: Crossovers are suppressed in windows with high heterozygosity. *a) Recombination suppression is observed in a stretch of high heterozygous windows in an otherwise actively recombining region at the end of chromosome IV. Mean heterozygous SNP counts in 5kb sliding windows (black) along with standard deviation among individuals (grey) are plotted for males (top panel) and females (bottom panel). Crossover counts in corresponding windows are overlaid (green: males, orange: females). Crossover suppressed regions with high heterozygosity are marked with horizontal red lines. b) Histogram of heterozygosity in crossover-containing 5kb windows shows that both male (green) and female (orange) crossovers (COs) mostly occur in windows with less than 50 heterozygous SNP per 5kb. Crossovers are rarely observed in windows with high heterozygosity (zoomed in region).*

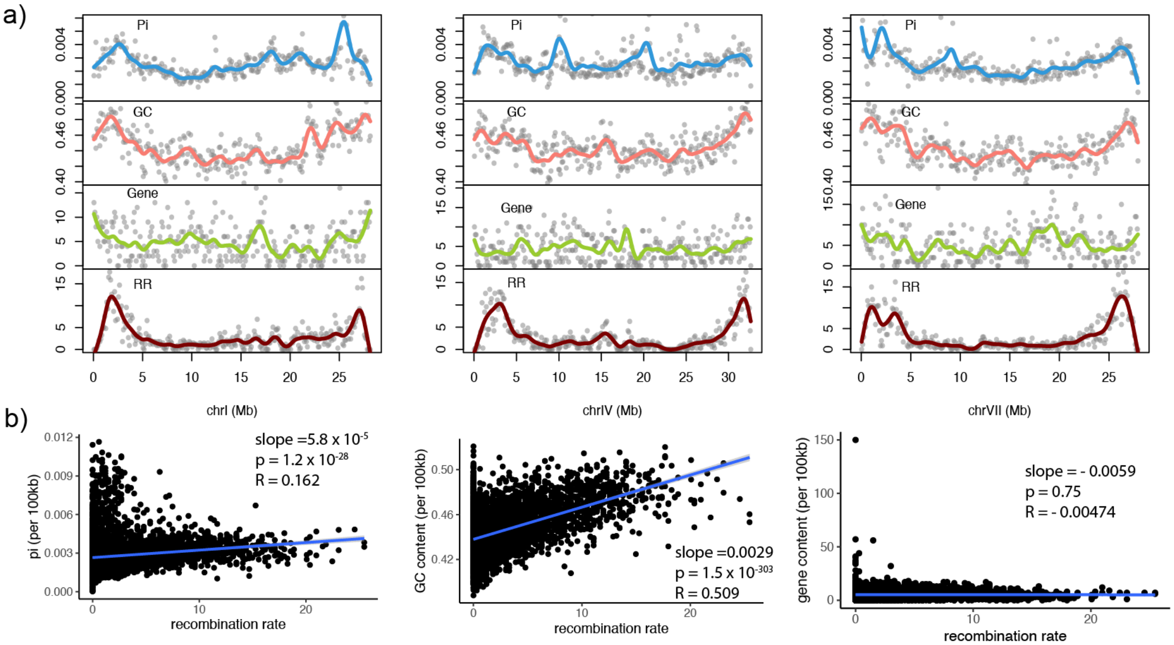

Supplementary Figure 8: Recombination rate is positively correlated with GC content and nucleotide diversity but not gene density. *a) Recombination rate (RR), GC content, and nucleotide diversity (pi) measured in 100kb sliding windows across three chromosomes (chrI, chrIV, and chrVII) shows a sub-telomeric enrichment. However, gene distribution does not show any obvious pattern and is mostly chromosome specific. b) Linear regressions of recombination rate versus nucleotide diversity (pi) and GC content across genome in 100kb windows show a significant positive relationship (pi:$\beta=5.832 \times 10^{-5} \pm 5.217 \times 10\text{-}6SE$, $F_{1,4643}=125$, $p<2 \times 10^{-16}$, GC: $\beta=2.866 \times 10^{-3} \pm 7.126 \times 10^{-6}SE$, $F_{1,4620}=1618$, $p<2 \times 10^{-16}$ respectively) whereas the negative correlation observed between recombination rate and gene density is not statistically significant at this scale (gene content: $\beta=-0.006 \pm 0.018SE$, $F_{1,4643}=0.1042$, $p=0.747$).*

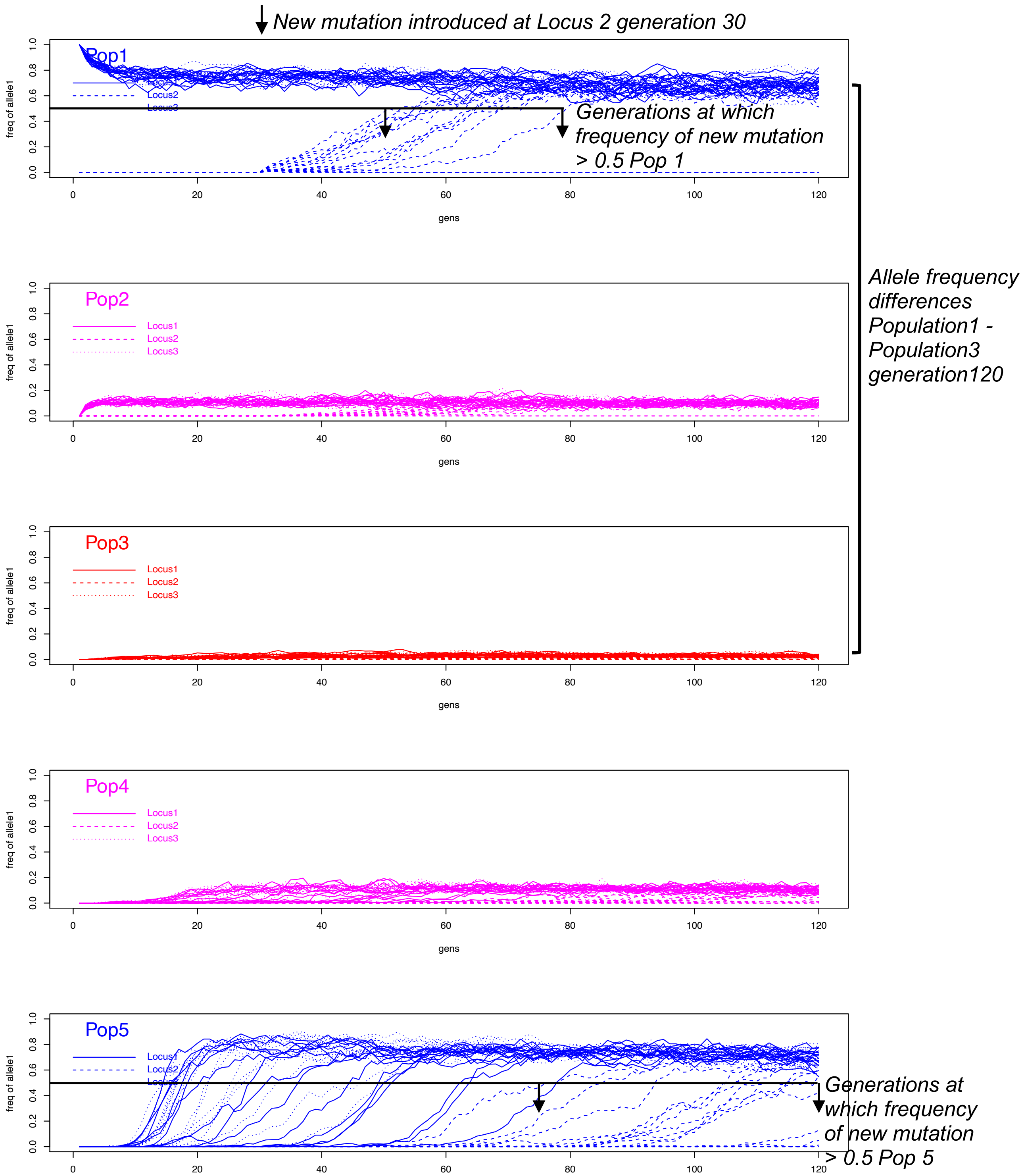

Supplementary Figure 9. **Forward simulations of three linked loci quantify the rate of the spread of an adaptive allele through hybrid zones maintained in selection migration balance under differing levels of heterochiasmy, recombination suppression, and overall recombination rate.** ***(Simulation1 – No-heterochiasmy, no recombination suppression, medium recombination rate).*** *Divergence between marine and freshwater populations is maintained, despite high rates of gene flow between populations. A new freshwater adaptive mutation is introduced at locus2 in generation 30. Each plot shows the frequency of the freshwater adaptive allele in populations 1 (top) through to 5 (bottom). Colors correspond to the habitats each with different simulated fitness effects of alleles (freshwater – blue; intertidal – magenta; marine – red).* See Supplementary Tables S8 and Table S9 and Supplementary methods for more information.

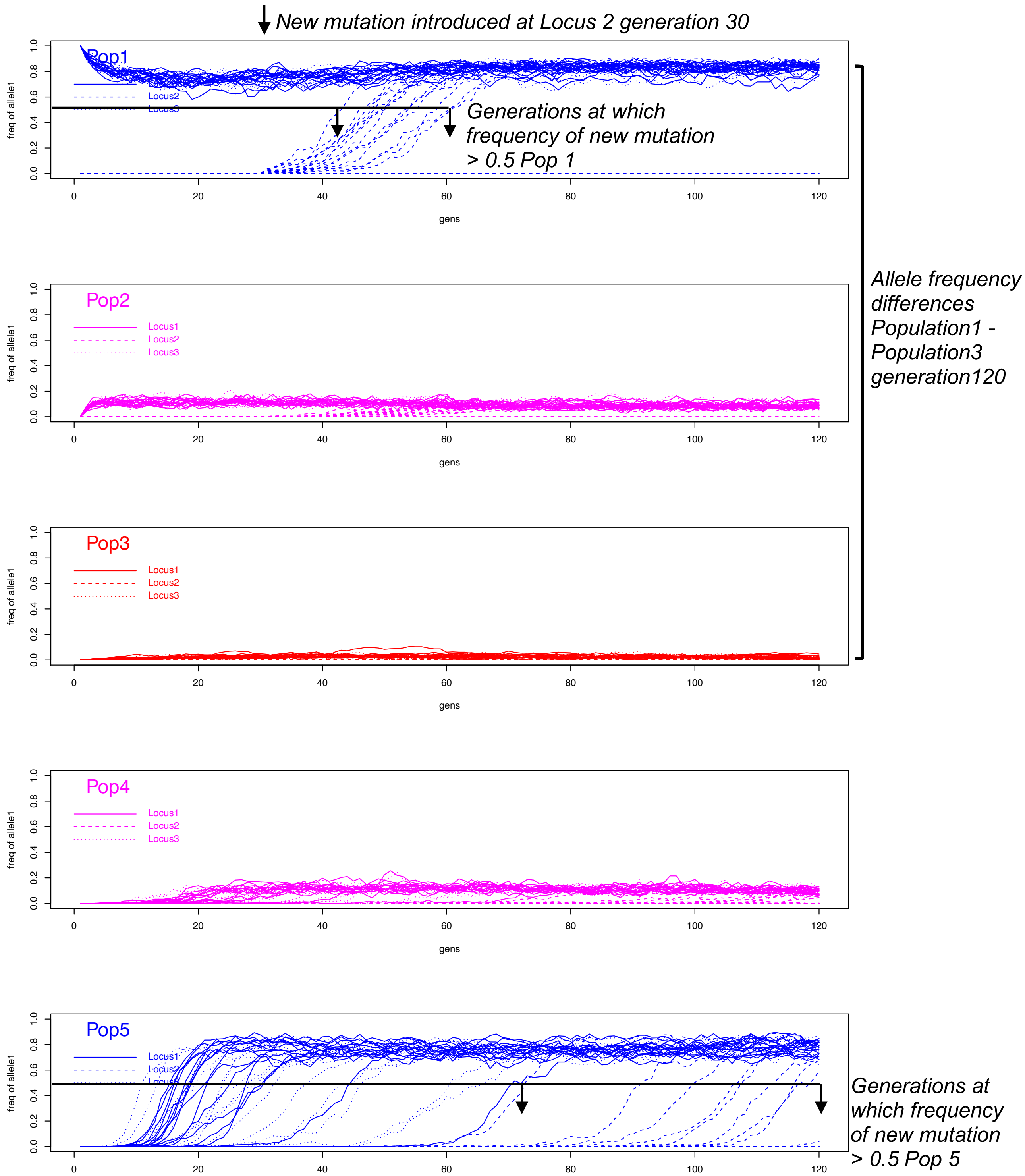

Supplementary Figure 10 Forward simulations of three linked loci quantify the rate of the spread of an adaptive allele through hybrid zones maintained in selection migration balance under differing levels of heterochiasmy, recombination suppression, and overall recombination rate. (Simulation2 – No-heterochiasmy, 10fold recombination suppression, medium recombination rate). *Divergence between marine and freshwater populations is maintained, despite high rates of gene flow between populations. A new freshwater adaptive mutation is introduced at locus2 in generation 30. Each plot shows the frequency of the freshwater adaptive allele in populations 1 (top) through to 5 (bottom). Colors correspond to the habitats each with different simulated fitness effects of alleles (freshwater – blue; intertidal – magenta; marine – red). See Supplementary Tables S8 and Table S9 and Supplementary methods for more information.*

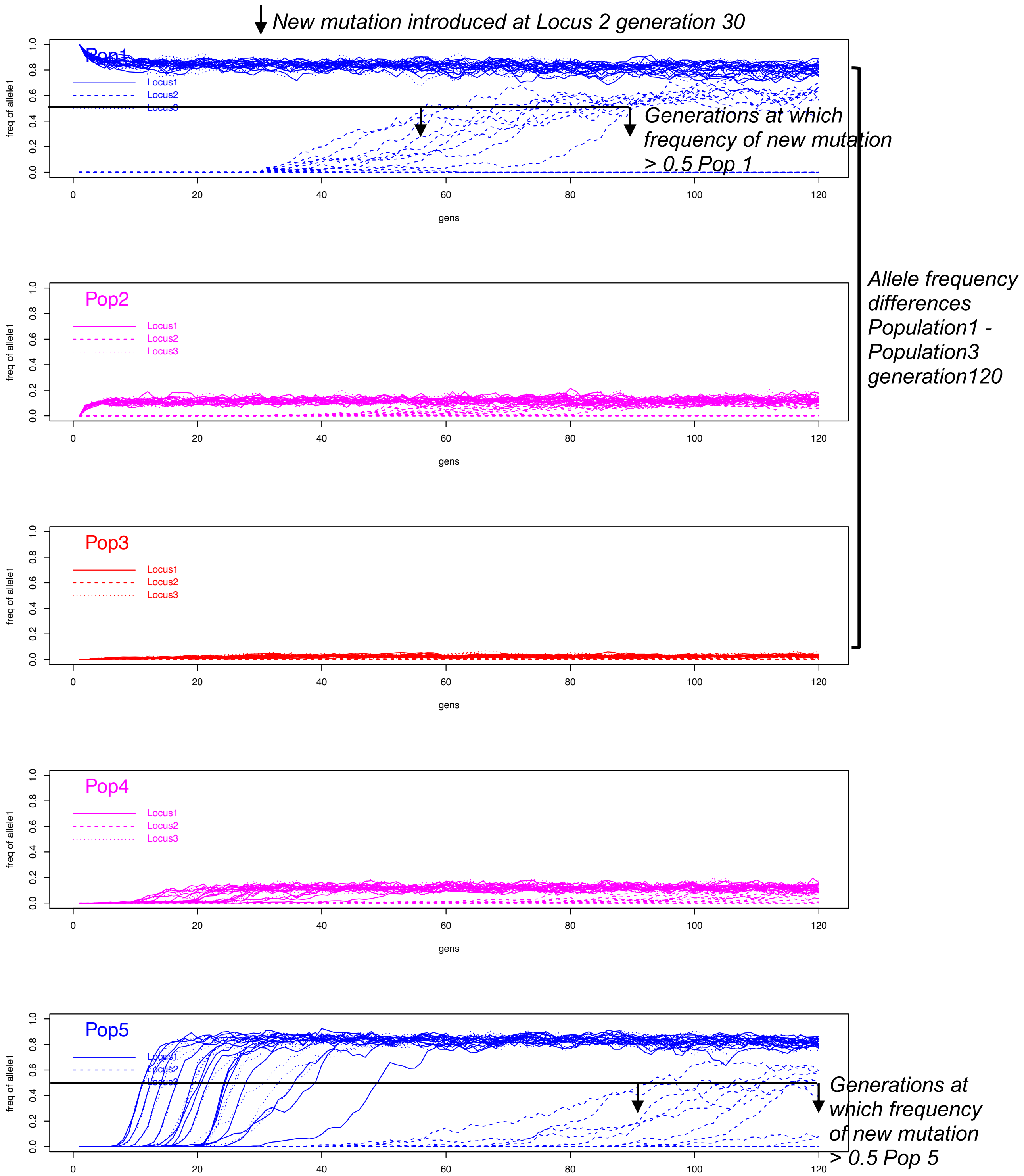

Supplementary Figure 11 Forward simulations of three linked loci quantify the rate of the spread of an adaptive allele through hybrid zones maintained in selection migration balance under differing levels of heterochiasmy, recombination suppression, and overall recombination rate. (Simulation3 – Strong heterochiasmy, no recombination suppression, medium recombination rate). *Divergence between marine and freshwater populations is maintained, despite high rates of gene flow between populations. A new freshwater adaptive mutation is introduced at locus2 in generation 30. Each plot shows the frequency of the freshwater adaptive allele in populations 1 (top) through to 5 (bottom). Colors correspond to the habitats each with different simulated fitness effects of alleles (freshwater – blue; intertidal – magenta; marine – red). See Supplementary Tables S8 and Table S9 and Supplementary methods for more information.*

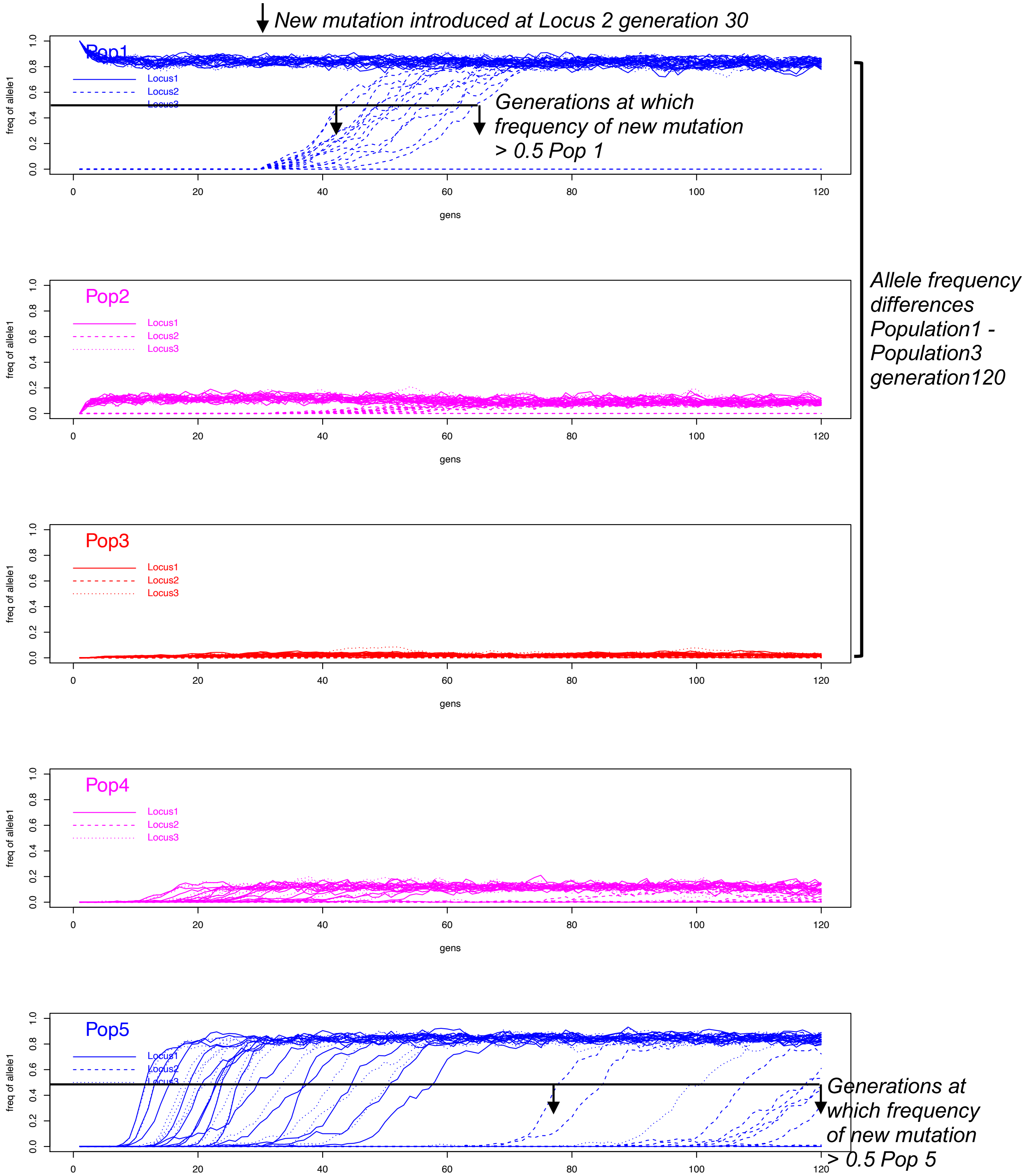

Supplementary Figure 12 Forward simulations of three linked loci quantify the rate of the spread of an adaptive allele through hybrid zones maintained in selection migration balance under differing levels of heterochiasmy, recombination suppression, and overall recombination rate. (Simulation4 – Strong heterochiasmy, 10fold recombination suppression, medium recombination rate). *Divergence between marine and freshwater populations is maintained, despite high rates of gene flow between populations. A new freshwater adaptive mutation is introduced at locus2 in generation 30. Each plot shows the frequency of the freshwater adaptive allele in populations 1 (top) through to 5 (bottom). Colors correspond to the habitats each with different simulated fitness effects of alleles (freshwater – blue; intertidal – magenta; marine – red). See Supplementary Tables S8 and Table S9 and Supplementary methods for more information.*

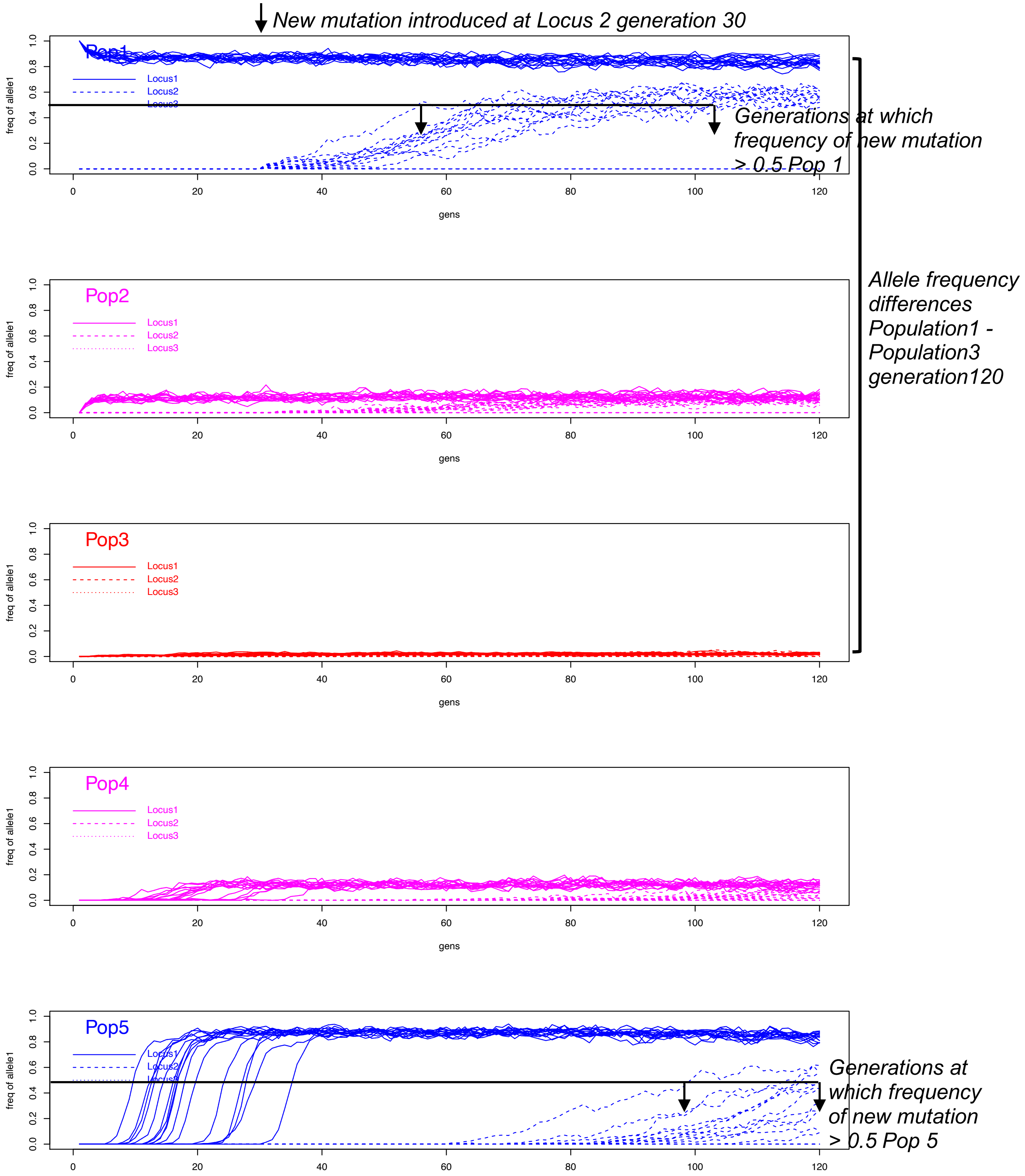

Supplementary Figure 13 Forward simulations of three linked loci quantify the rate of the spread of an adaptive allele through hybrid zones maintained in selection migration balance under differing levels of heterochiasmy, recombination suppression, and overall recombination rate. (Simulation5 – No heterochiasmy, no recombination suppression, high recombination rate). *Divergence between marine and freshwater populations is maintained, despite high rates of gene flow between populations. A new freshwater adaptive mutation is introduced at locus2 in generation 30. Each plot shows the frequency of the freshwater adaptive allele in populations 1 (top) through to 5 (bottom). Colors correspond to the habitats each with different simulated fitness effects of alleles (freshwater – blue; intertidal – magenta; marine – red). See Supplementary Tables S8 and Table S9 and Supplementary methods for more information.*

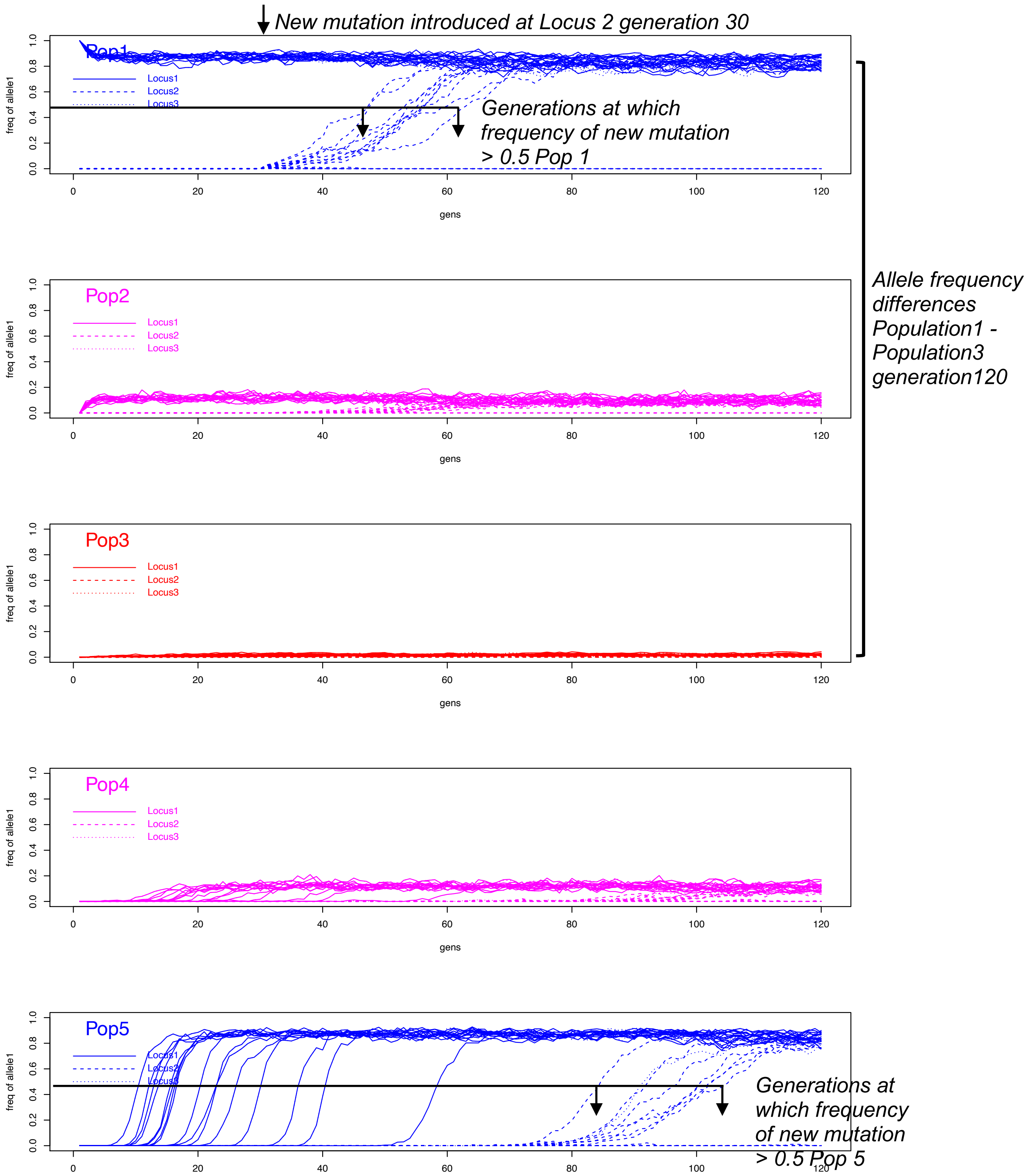

Supplementary Figure 14 Forward simulations of three linked loci quantify the rate of the spread of an adaptive allele through hybrid zones maintained in selection migration balance under differing levels of heterochiasmy, recombination suppression, and overall recombination rate. (Simulation6 – No heterochiasmy, 10fold recombination suppression, high recombination rate). *Divergence between marine and freshwater populations is maintained, despite high rates of gene flow between populations. A new freshwater adaptive mutation is introduced at locus2 in generation 30. Each plot shows the frequency of the freshwater adaptive allele in populations 1 (top) through to 5 (bottom). Colors correspond to the habitats each with different simulated fitness effects of alleles (freshwater – blue; intertidal – magenta; marine – red). See Supplementary Tables S8 and Table S9 and Supplementary methods for more information.*

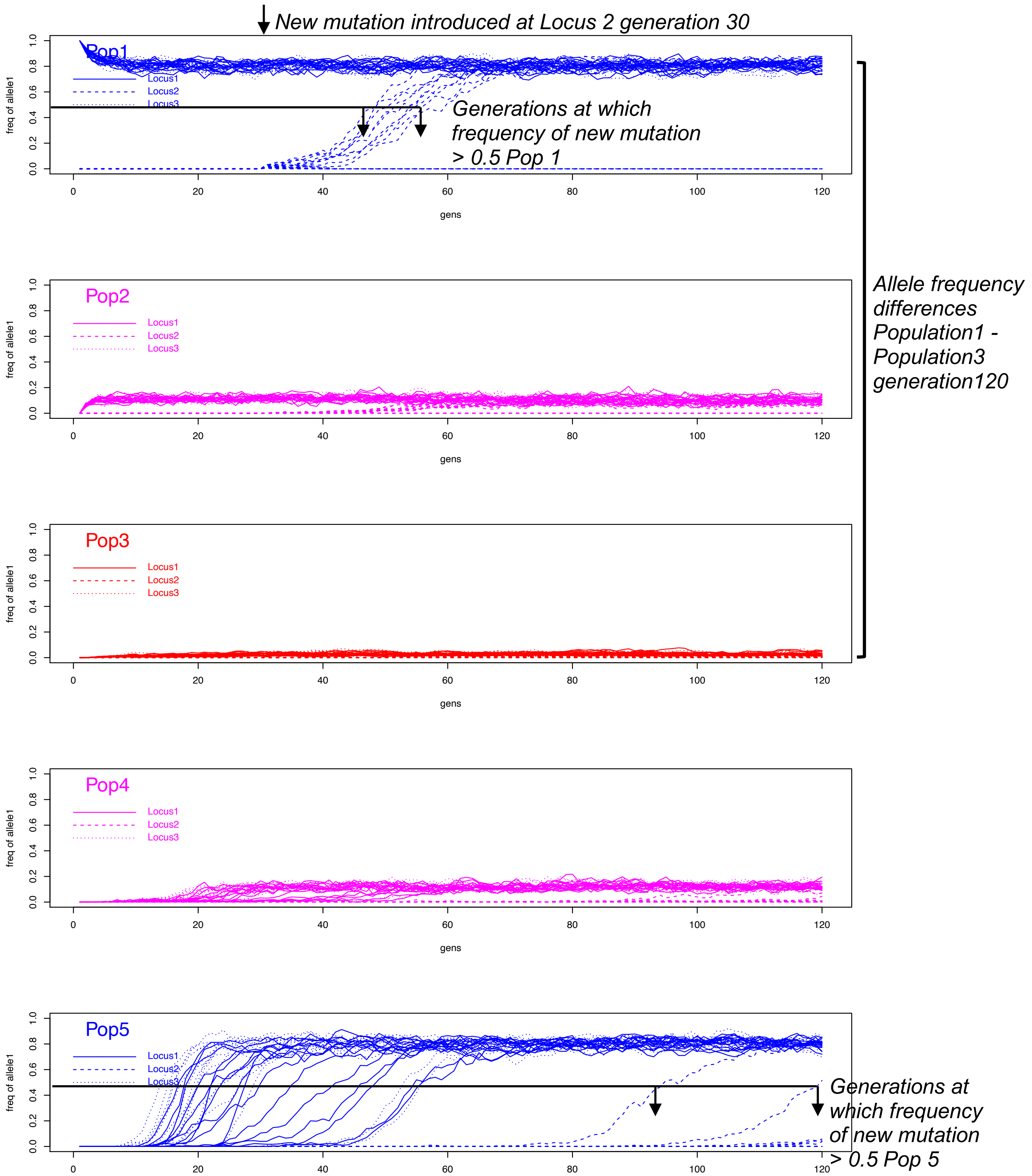

Supplementary Figure 15 Forward simulations of three linked loci quantify the rate of the spread of an adaptive allele through hybrid zones maintained in selection migration balance under differing levels of heterochiasmy, recombination suppression, and overall recombination rate. (Simulation7 – No heterochiasmy, no recombination suppression, low recombination rate). *Divergence between marine and freshwater populations is maintained, despite high rates of gene flow between populations. A new freshwater adaptive mutation is introduced at locus2 in generation 30. Each plot shows the frequency of the freshwater adaptive allele in populations 1 (top) through to 5 (bottom). Colors correspond to the habitats each with different simulated fitness effects of alleles (freshwater – blue; intertidal – magenta; marine – red). See Supplementary Tables S8, Tables S9 and Supplementary methods for more information.*

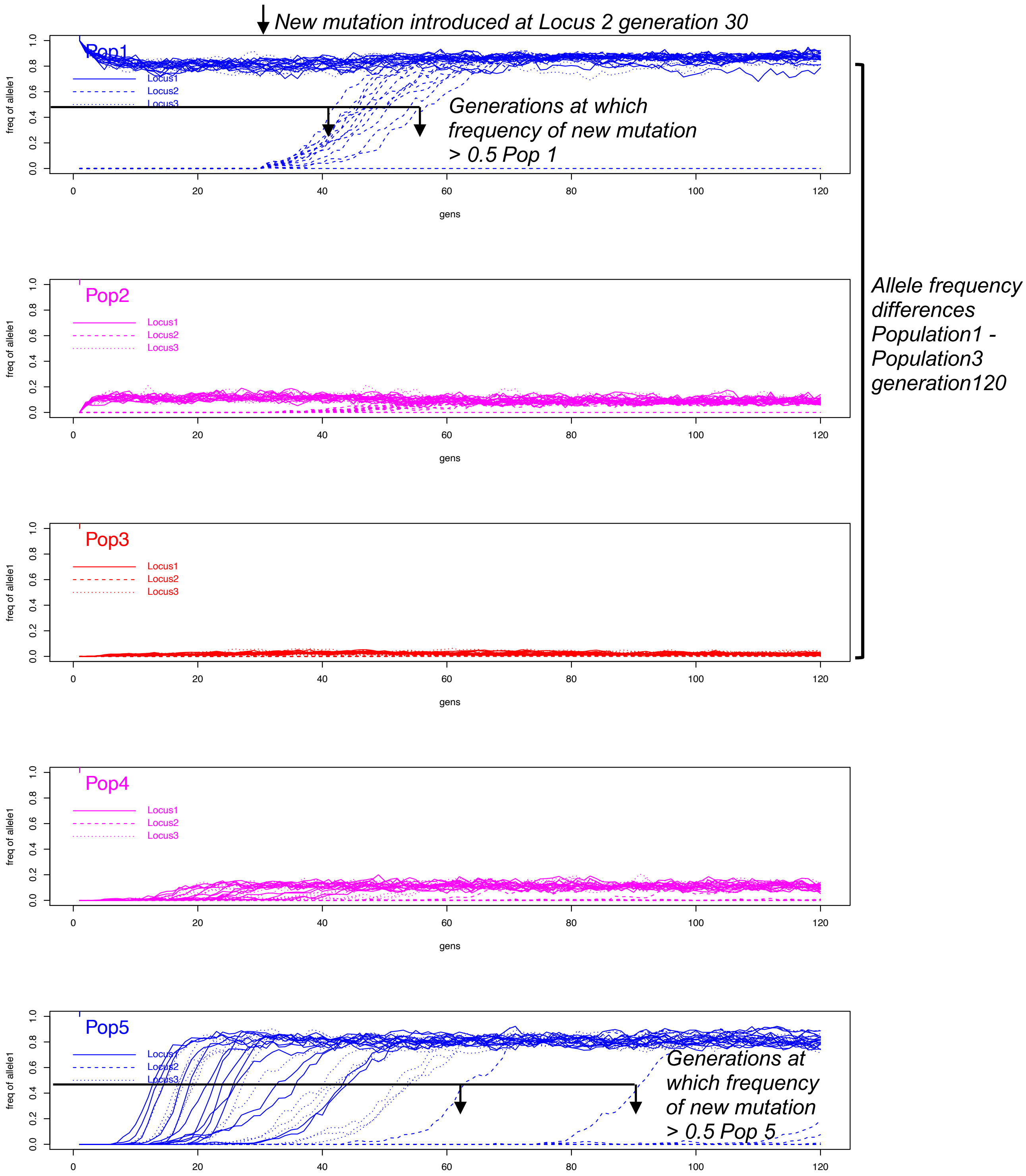

Supplementary Figure 16 Forward simulations of three linked loci quantify the rate of the spread of an adaptive allele through hybrid zones maintained in selection migration balance under differing levels of heterochiasmy, recombination suppression, and overall recombination rate. (Simulation8 – No heterochiasmy, 10fold recombination suppression, low recombination rate). *Divergence between marine and freshwater populations is maintained, despite high rates of gene flow between populations. A new freshwater adaptive mutation is introduced at locus2 in generation 30. Each plot shows the frequency of the freshwater adaptive allele in populations 1 (top) through to 5 (bottom). Colors correspond to the habitats each with different simulated fitness effects of alleles (freshwater – blue; intertidal – magenta; marine – red). See Supplementary Tables S8 and Table S9 and Supplementary methods for more information.*

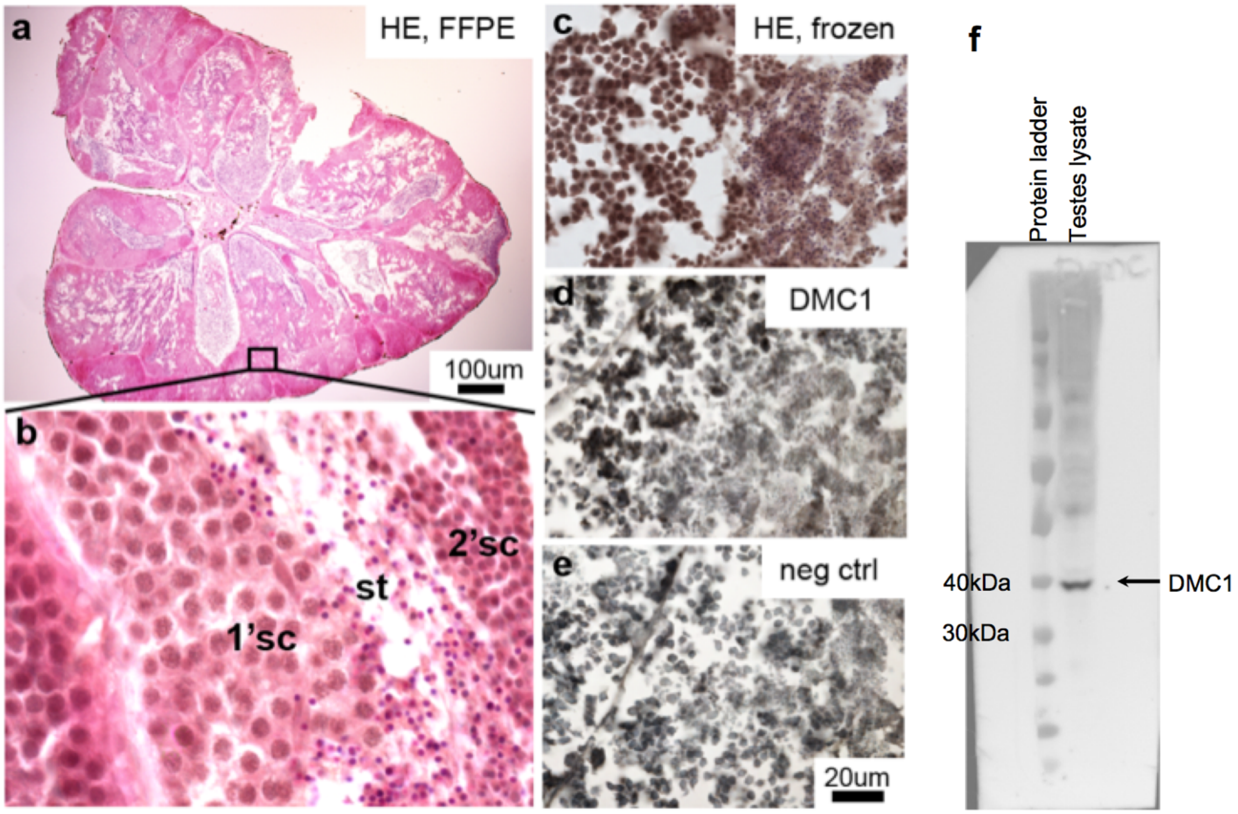

Supplementary Figure 17 Enrichment for primary spermatocytes in meiosis I phase. *a) The enrichment of primary spermatocytes was estimated from Haematoxylin-Eosin (HE) stained Formalin-Fixed Paraffin Embedded (FFPE) testes sections. b) Zoom-in illustrating primary spermatocytes (1'sc), secondary spermatocytes (2'sc) and spermatids (st). c, d and e) Verification of anti-DMC1 antibody specificity on primary spermatocytes, done on consecutive sections of frozen testes samples. DMC1 signal (d) is specific to primary spermatocytes on the left side of the panel (darker grey/black staining). f) Western Blot on meiocyte-enriched testes with anti-DMC1 showing a band of expected DMC1 size. All experiments were repeated on multiple different testes and yielded qualitatively similar results.*

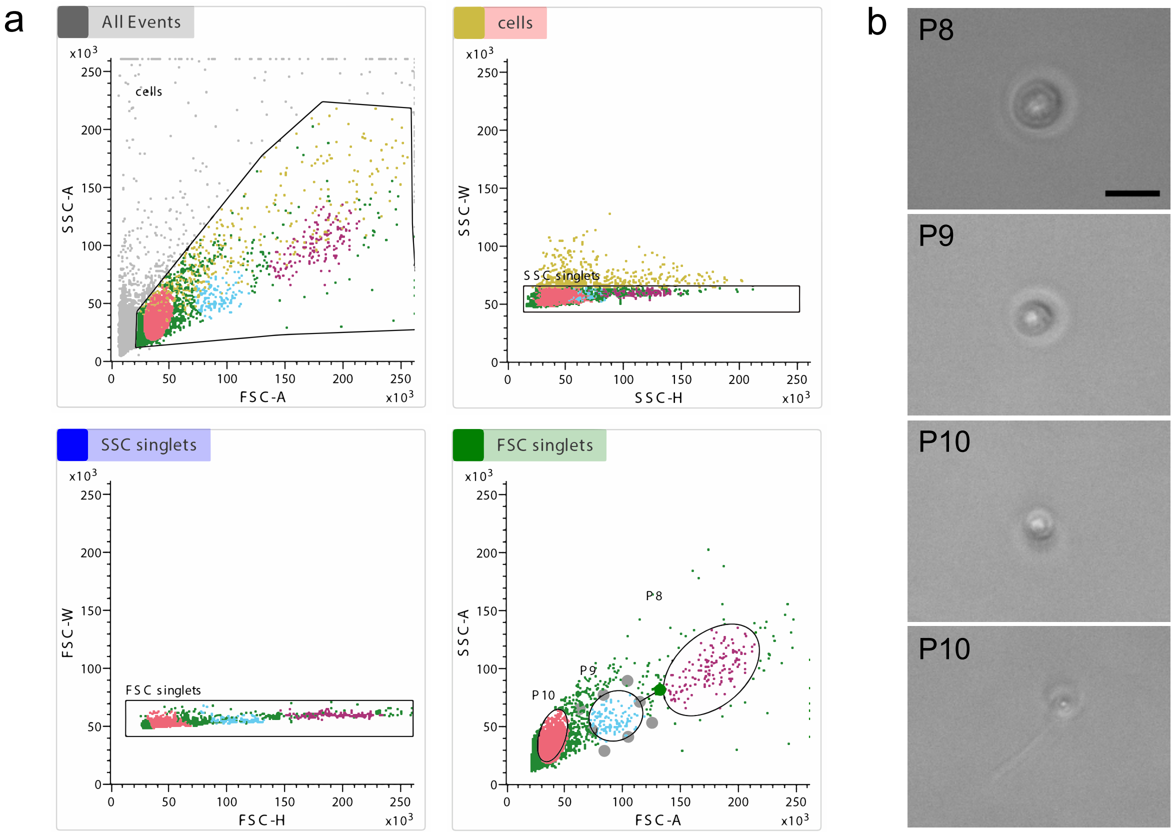

Supplementary Figure 18 Sorting of primary spermatocytes for chromatin accessibility profiling via ATAC-seq. *a) Gating strategy, P8 = primary spermatocytes, P9 = secondary spermatocytes, P10 = spermatids and sperm. Only P8 were used in ATAC-seq. b) Representative photos of cell populations from >100-cell subsamples of sorted cells.. Scale bar 10um. each meiotic stage for cells sorted for subsequent ATAC-sequencing.*

Family	Ecotype	Number of offspring	Dad coverage (x)	Mum coverage (x)	Offspring mean coverage (x) ± SD	Dad informative SNP count	Mum informative SNP count	Dad inter-SNP mean distance (bp)	Mum inter-SNP mean distance (bp)
**X1**	Freshwater	94	38.27	49.43	13.18 ± 3.50	259704	250250	1541	1591
**X4**	Freshwater	93	70.54	54.69	19.83 ± 6.21	385432	434153	1038	922
**X268**	Freshwater	92	236.14	188.56	14.11 ± 3.60	441892	282717	906	1406
**X284**	Freshwater	91	58.65	80.06	11.86 ± 2.56	320492	173594	1247	2301
**X350**	Freshwater	93	60.98	67.45	13.35 ± 2.44	285827	238058	1401	1679
**X351**	Freshwater	93	229.45	298.05	13.38 ± 2.68	350647	283794	1141	1410
**X11**	Marine	94	77.81	63.65	11.65 ± 2.17	385709	319840	1039	1252
**X20**	Marine	91	57.69	138.48	14.07 ± 5.48	325633	307571	1230	1300
**X291**	Marine	94	57.58	70.09	12.46 ± 2.97	465765	364930	860	1098
**X294**	Marine	94	119.55	117.69	14.11 ± 2.99	492870	410454	813	976
**X295**	Marine	93	35.04	44.24	13.24 ± 3.60	216217	111255	1851	3586
**X296**	Marine	93	56.15	68.44	13.62 ± 2.89	465613	246504	861	1625
**X273**	Hybrid	94	62.47	64.30	13.45 ± 2.71	461511	345952	868	1155
**X274**	Hybrid	94	87.67	72.32	13.63 ± 2.99	451754	312601	887	1282
**X800**	Hybrid	94	32.82	45.05	13.32 ± 2.84	269782	323189	1477	1239
**X366**	Hybrid	86	69.86	59.70	13.36 ± 3.68	474780	329376	844	1216
**X389**	Hybrid	93	62.58	69.91	14.63 ± 2.35	513093	385061	781	1041
**X391**	Hybrid	93	63.70	57.80	14.57 ± 2.68	501055	367392	798	1090

***Supplementary Table 1** : Family, sequencing, SNP summary table*

**merged divergent hotspot region**	**chr**	**start**	**stop**	**Marine CO Count[1]**	**Freshwater CO Count[1]**	**Hybrid CO Count[1]**	**Direction of Divergence in CO Counts**
chrXII:1333868-1339417	chrXII	1333868	1339417	3	8	2	Freshwater>Hybrid
chrXVIII:2579793-2584793	chrXVIII	2579793	2584793	1	5	3	Freshwater>Marine
chrXVIII:14592056-14597056	chrXVIII	14592056	14597056	1	5	4	Freshwater>Marine
chrX:13823856-13828856	chrX	13823856	13828856	0	6	1	Freshwater>Marine; Freshwater>Hybrid
chrXIII:18523940-18529348	chrXIII	18523940	18529348	1	7	2	Freshwater>Marine; Freshwater>Hybrid
chrXXI:11393230-11406225	chrXXI	11393230	11406225	9	17	5	Freshwater>Marine; Freshwater>Hybrid
chrI:1543413-1554456	chrI	1543413	1554456	7	5	3	Marine>Freshwater; Freshwater>Marine; Marine>Hybrid[a]
chrIII:16405102-16412742	chrIII	16405102	16412742	5	1	7	Hybrid>Freshwater
chrVII:2397086-2404154	chrVII	2397086	2404154	0	1	7	Hybrid>Marine
chrXIX:1449114-1455016	chrXIX	1449114	1455016	2	2	6	Hybrid>Marine
chrXI:14097150-14102150	chrXI	14097150	14102150	7	1	3	Marine>Freshwater; Marine>Hybrid
chrXIX:1623924-1631003	chrXIX	1623924	1631003	10	4	3	Marine>Hybrid

1 Counts of all crossover midpoints (not downsampled, but excluding crossovers that overlap scaffold boundaries and/or have interval resolution greater than 10kb) that fall within the merged genomic region are shown for each ecotype. a Neighbouring 5kb windows within this region show crossover counts with freshwater>marine, marine>freshwater, and marine>hybrids

Supplementary Table 2*: Genomic regions containing 5kb hotspot windows with >=6 total crossovers and significant differences in crossover counts between marine, freshwater and hybrid fish. Statistical tests were performed in 5kb intervals on equal numbers of crossovers from all three ecotypes (see Tables S3-S5), and merged to generate intervals shown in this table. Assuming a uniform distribution, the probability 6 of 18926 crossovers falling within 5kb of each other by chance is* $1.6x10^{-30}$.

Freshwater Crossover 5kb Window [1]	chr	start	stop	Freshwater Crossover Count	Marine Crossover Count	Hybrid Crossover Count	log2FoldChange [Marine Crossover Count / Freshwater Crossover Count] [2]	log2FoldChange [Hybrid Crossover Count / Freshwater Crossover Count] [2]	significant_contrast
chrI:1547951-1552951	chrI	1547951	1552951	1	5	1	**2.21299**	0	Freshwater_v_Marine
chrIII:16407356-16412356	chrIII	16407356	16412356	1	3	6	1.49476	**2.47131**	Freshwater_v_Hybrid
chrX:13823856-13828856	chrX	13823856	13828856	6	0	0	**-5.93074**	**-5.93074**	Freshwater_v_Marine; Freshwater_v_Hybrid
chrXII:1333868-1338868	chrXII	1333868	1338868	7	3	0	-1.19555	**-6.14975**	Freshwater_v_Hybrid
chrXII:1333976-1338976	chrXII	1333976	1338976	6	3	0	-0.976541	**-5.93074**	Freshwater_v_Hybrid
chrXII:1334417-1339417	chrXII	1334417	1339417	6	2	0	-1.53842	**-5.93074**	Freshwater_v_Hybrid
chrXIII:18523940-18528940	chrXIII	18523940	18528940	6	1	2	**-2.47131**	-1.53842	Freshwater_v_Marine
chrXIII:18524348-18529348	chrXIII	18524348	18529348	6	1	1	**-2.47131**	**-2.47131**	Freshwater_v_Marine; Freshwater_v_Hybrid
chrXXI:11393230-11398230	chrXXI	11393230	11398230	9	4	2	-1.15024	**-2.11548**	Freshwater_v_Hybrid
chrXXI:11397018-11402018	chrXXI	11397018	11402018	6	1	3	**-2.47131**	-0.976541	Freshwater_v_Marine
chrXXI:11400304-11405304	chrXXI	11400304	11405304	8	3	0	-1.38565	**-6.33985**	Freshwater_v_Hybrid
chrXXI:11401020-11406020	chrXXI	11401020	11406020	7	3	0	-1.19555	**-6.14975**	Freshwater_v_Hybrid
chrXXI:11401225-11406225	chrXXI	11401225	11406225	6	3	0	-0.976541	**-5.93074**	Freshwater_v_Hybrid

1 Freshwater 5kb crossover hotspot windows with significant fold change difference in crossover counts relative to marine and hybrid ecotypes (FDR <0.05) and >=6 total crossovers in the focal window. Assuming a uniform distribution, the probability of this happening by chance (6 of 18926 crossovers falling within 5kb of each other) is 1.6x10^-30.
2 log2FoldChange q<0.05 shown in bold. Pseudocount of 0.1 added to both sides of log ratio.

Supplementary Table 3 *: 5kb windows with significant differences in crossover counts between freshwater and marine fish and freshwater and hybrid fish. For each focal window with >=6 total crossovers among the ecotypes being compared, down sampled crossover counts for all three ecotypes are shown with fold changes ratios with qvalue <0.05 shown in bold. Only crossovers not spanning scaffold boundaries, and with crossover interval resolution <=10000bp, down sampled to generate equal numbers of crossovers per ecotype were included in the analysis.*

Marine Crossover 5kb Window [1]	chr	start	stop	Marine CO Count	Freshwater CO Count	Hybrid CO Count	log2FoldChange [Freshwater CO Count / Marine CO Count] [2]	log2FoldChange [Hybrid CO Count / Marine CO Count] [2]	significant_contrast
chrI:1543413-1548413	chrI	1543413	1548413	1	5	2	**2.21299**	0.932886	Marine_v_Freshwater
chrXI:14097150-14102150	chrXI	14097150	14102150	6	1	1	**-2.47131**	**-2.47131**	Marine_v_Freshwater; Marine_v_Hybrid
chrXIX:1449114-1454114	chrXIX	1449114	1454114	2	2	6	0	**1.53842**	Marine_v_Hybrid
chrXIX:1624328-1629328	chrXIX	1624328	1629328	8	4	1	-0.982298	**-2.88042**	Marine_v_Hybrid
chrXIX:1625740-1630740	chrXIX	1625740	1630740	8	3	0	-1.38565	**-6.33985**	Marine_v_Hybrid
chrXIX:1626003-1631003	chrXIX	1626003	1631003	7	3	0	-1.19555	**-6.14975**	Marine_v_Hybrid
chrXVIII:2579793-2584793	chrXVIII	2579793	2584793	1	5	2	**2.21299**	0.932886	Marine_v_Freshwater
chrXVIII:14592056-14597056	chrXVIII	14592056	14597056	1	5	2	**2.21299**	0.932886	Marine_v_Freshwater

1 Marine 5kb crossover hotspot windows with significant fold change difference in crossover counts relative to freshwater and hybrid ecotypes (FDR <0.05) and >=6 total crossovers in the focal window. Assuming a uniform distribution, the probability of this happening by chance (6 of 18926 crossovers falling within 5kb of each other) is 1.6x10^-30.
2 log2FoldChange q<0.05 shown in bold

Supplementary Table 4*: 5kb windows with significant differences in crossover counts between marine and freshwater fish and marine and hybrid fish. For each focal window with >=6 total crossovers among the ecotypes being compared, down sampled crossover counts for all three ecotypes are shown with fold changes ratios with qvalue <0.05 shown in bold. Only crossovers not spanning scaffold boundaries, and with crossover interval resolution <=10000bp, down sampled to generate equal numbers of crossovers per ecotype were included in the analysis.*

Hybrid Crossover 5kb Window [1]	chr	start	stop	Hybrid CO Count	Marine CO Count	Freshwater CO Count	log2FoldChange [Marine CO Count / Hybrid CO Count] [2]	log2FoldChange [Freshwater CO Count / Hybrid CO Count] [2]	significant_contrast
chrI:1549456-1554456	chrI	1549456	1554456	1	5	0	**2.21299**	-3.45943	Hybrid_v_Marine
chrIII:16405102-16410102	chrIII	16405102	16410102	7	4	1	-0.792195	**-2.69032**	Hybrid_v_Freshwater
chrIII:16407742-16412742	chrIII	16407742	16412742	6	2	0	-1.53842	**-5.93074**	Hybrid_v_Freshwater
chrVII:2397086-2402086	chrVII	2397086	2402086	6	0	1	**-5.93074**	-2.47131	Hybrid_v_Marine
chrVII:2399154-2404154	chrVII	2399154	2404154	6	0	1	**-5.93074**	-2.47131	Hybrid_v_Marine
chrXIX:1450016-1455016	chrXIX	1450016	1455016	6	1	2	**-2.47131**	-1.53842	Hybrid_v_Marine
chrXIX:1623924-1628924	chrXIX	1623924	1628924	2	8	4	**1.94753**	0.965235	Hybrid_v_Marine
chrXIX:1625364-1630364	chrXIX	1625364	1630364	1	7	3	**2.69032**	1.49476	Hybrid_v_Marine
chrXXI:11400166-11405166	chrXXI	11400166	11405166	2	3	8	0.561879	**1.94753**	Hybrid_v_Freshwater
chrXXI:11400197-11405197	chrXXI	11400197	11405197	1	3	8	1.49476	**2.88042**	Hybrid_v_Freshwater

1 Hybrid 5kb crossover hotspot windows with significant fold change difference in crossover counts relative to freshwater and marine ecotypes (FDR <0.05) and >=6 total crossovers in the focal window. Assuming a uniform distribution, the probability of this happening by chance (6 of 18926 crossovers falling within 5kb of each other) is 1.6x10^-30.
2 log2FoldChange q<0.05 shown in bold

Supplementary Table 5*: 5kb windows with significant differences in crossover counts between hybrid and freshwater fish and hybrid and marine fish. For each focal window with >=6 total crossovers among the ecotypes being compared, down sampled crossover counts for all three ecotypes are shown with fold changes ratios with qvalue <0.05 shown in bold. Only crossovers not spanning scaffold boundaries, and with crossover interval resolution <=10000bp, down sampled to generate equal numbers of crossovers per ecotype were included in the analysis.*

Male			Female		
Motif	Probability	Frequency at CO sites	Motif	Probability	Frequency at CO sites
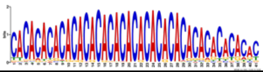	3.1e-090	25.5%	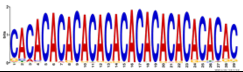	2.4e-139	23.2%
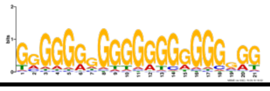	9.4e-041	51.3%	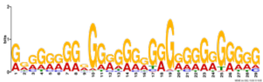	6.8e-028	39.2%
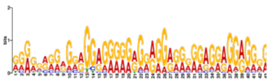	2.3e-027	1.6%	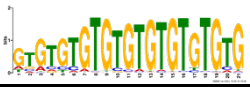	5.0e-019	33.6%

Supplementary Table 6 *: Motifs discovered using MEME considering all male and female high-resolution (<5kb) crossover intervals. Probability corresponds to the E-value returned by MEME (an estimate of the expected number of motifs with the given log likelihood ratio (or higher), and with the same width and site count, that one would find in a similarly sized set of random sequences).*

Sex	Inversion at	Inversion detected families	Ecotype
**Male**	chrI: 25,264,236 - 25,720,158	X294	Marine
	chrII: 22,372,205 - 23,174,871	X268, X294, X296, X350, X351, X366, X389, X391	Marine, FW, Hybrid B
	chrIX: 5,655,297 - 7,950,866	X389	Hybrid B
	chrXI: 5,431,984 - 5,868,073	X291	Marine
	chrXI: 15,730,574 - 16,638,140	X11, X20, X268, X284, X291, X294, X296, X366, X391	Marine, FW, Hybrid B
	chrXVI: 17,195,676 - 17,968,133	X11, X20, X268, X273, X274, X284, X294, X296, X350, X351, X366, X389, X4, X800	Marine, FW, Hybrid B, Hybrid A
	chrXVII: 641211 - 769373	X391	Hybrid B
	chrXXI : 5,681,441 - 7,787,895	X1, X11, X284, X294, X296, X350, X351, X366, X389, X4, X800	Marine, FW, Hybrid B, Hybrid A
**Female**	chrI: 25,267,445 - 25,725,739	X11, X291	Marine
	chrII: 22,358,302 - 23,062,538	X1, X268, X366, X800	Marine, FW, Hybrid A, Hybrid B
	chrIX : 5,647,525 - 7,282,910	X11, X366	Marine, Hybrid B
	chrXI : 5431082 - 5858743	X11, X20, X294, X389, X800	Marine, Hybrid A, Hybrid B
	chrXI: 15,734,076 - 16,500,546	X1, X11, X273, X296, X800	Marine, FW, Hybrid A,
	chrXVI: 15,870,586 - 17,836,051	X1, X11, X273, X20, X274, X284, X291, X294, X296, X350, X351, X366, X389, X391, X4, X800	Marine, FW, Hybrid A, Hybrid B
	chrXVII: 641353 - 769480	X350	FW
	chrXXI: 5,726,992 -7,494,035	X11, X268, X273, X284, X291, X294, X295, X296, X350,351, X366, X389, X391, X800	Marine, FW, Hybrid A, Hybrid B
***Hybrid A: (FW x Marine)F1; Hybrid B: (Mar x FW)F1**			

Supplementary Table 7*: Details of chromosomal inversions (compared to reference genome) detected in this data set*

simulation	recombination rate female (cM)	recombination rate male (cM)	effective sex-averaged recombination rate	recombination suppression in cis(coupled)-heterozygotes	Population 1 mean+/-SD generation to reach frequency 0.5	Population 1 probability of reaching frequency 0.5 within 120 generations	Population 5 mean+/-SD generation to reach frequency 0.5	Population 5 probability of reaching frequency 0.5 within 120 generations	Locus 1 Mean allele frequency difference at generation 120 between freshwater population 1 and marine population 3 (Δp +/- SD)	Locus 2 Mean allele frequency difference at generation 120 between freshwater population 1 and marine population 3 (Δp +/- SD)	Locus 3 Mean allele frequency difference at generation 120 between freshwater population 1 and marine population 3 (Δp +/- SD)	Figure	Description
1	0,55	0,55	0,55	none	62.7+/-7.4	0,8	102.7+/-13.9	0,6	0.66+/-0.06	0.49+/-0.25	0.65+/-0.06	Supp Fig S9	No-heterochiasmy, No recombination suppression, medium recombination rate
2	0,55	0,55	0,55	10fold	52.2+/-5.4	0,9	103.4+/-14.9	0,5	0.80+/-0.03	0.73+/-0.29	0.80+/-0.04	Supp Fig S10	No-heterochiasmy, 10fold recombination suppression, medium recombination rate
3	1	0,1	0,55	none	74.4+/-11.1	0,6	107.5+/-9.1	0,4	0.79+/-0.04	0.33+/-0.30	0.78+/-0.04	Supp Fig S11	Strong-heterochiasmy, No recombination suppression, medium recombination rate
4	1	0,1	0,55	10fold	53.2+/-7.0	0,8	101.8+/-18.8	0,3	0.81+/-0.02	0.68+/-0.34	0.81+/-0.02	Supp Fig S12	Strong-heterochiasmy, 10fold recombination suppression, medium recombination rate
5	1	1	1	none	79.6+/-12.9	0,8	111.0+/-9.6	0,2	0.81+/-0.04	0.41+/-0.25	0.81+/-0.04	Supp Fig S13	No-heterochiasmy, No recombination suppression, high recombination rate
6	1	1	1	10fold	55.7+/-5.1	0,6	98.4+/-7.3	0,4	0.80+/-0.05	0.51+/-0.40	0.82+/-0.03	Supp Fig S14	No-heterochiasmy, 10fold recombination suppression, high recombination rate
7	0,1	0,1	0,1	none	53.6+/-3.7	0,6	107.5+/-17.7	0,1	0.77+/-0.05	0.47+/-0.43	0.78+/-0.03	Supp Fig S15	No-heterochiasmy, No recombination suppression, low recombination rate
8	0,1	0,1	0,1	10fold	48.8+/-4.2	0,6	78.0+/-19.8	0,1	0.84+/-0.04	0.65+/-0.39	0.83+/-0.04	Supp Fig S16	No-heterochiasmy, 10fold recombination suppression, low recombination rate

***Supplementary Table 8**: Forward simulations quantify the rate of the spread of an adaptive allele through hybrid zones maintained in selection migration balance under differing levels of heterochiasmy, recombination suppression, and overall recombination rate. Summary statistics quantify the generation in which the adaptive allele frequency at Locus 2 reaches a frequency greater than 0.5 in Population1 and 5, the probability of this occurring within 120 generations (proportion of occurrences in all replicate runs), and the mean allele frequency difference +/- standard deviation that is maintained between freshwater population 1 and marine population 3 at each of the three loci at generation 120. Figure numbers corresponding to plots of allele frequency changes over time in each of the populations are shown for each of the simulation conditions in column 12.*

2-locus haplotype

L1 L3	Marine	Intertidal	Freshwater
M M	0.5	0.5	0.05
F M	0.225	0.25	0.225
M F	0.225	0.25	0.225
F F	0.05	0.5	0.5

3-locus haplotype

L1L2L3	Marine	Intertidal	Freshwater
MMM	0.5	0.25	0.05
FMM	0.35	0.125	0.2
MFM	0.35	0.125	0.2
MMF	0.35	0.125	0.2
FFM	0.2	0.125	0.35
FMF	0.2	0.125	0.35
MFF	0.2	0.125	0.35
FFF	0.05	0.25	0.5

Supplementary Table 9*: Fitness of simulated offspring was based on the additive effects of their multi-locus haplotypes (fitness effect hapotype1 + fitness effect haplotype 2), and the habitat in which they live. This number was used to assign survival probabilities to each offspring.*

Supplementary Methods

1) Investigating the effect of heterochiasmy and recombination on the spread of adaptive mutations through hybrid zones using forward simulations.

We used population genetic simulations to investigate how heterochiasmy and recombination suppression influence the spread of freshwater adaptive alleles to neighbouring freshwater populations via hybrid zones. Specifically we used a five population model to simulate a selection-migration balance maintaining divergence among a freshwater (population 1), intertidal (population 2), and marine population (population 3). A second hybrid zone with intertidal (population 4) and freshwater population (population 5) was included with connection to the first via marine population (population 3), resulting in parallel hybrid zones with access to the same marine population.

```
       Population1 <-> Population2 <-> Population3 <-> Population4 <-> Population5
       (freshwater)    (intertidal)      (marine)      (intertidal)    (freshwater)
Ne:        200             200             600             200             200


chromosome homolog1:          Locus1 ----- Locus2 ----- Locus3
                                      X            X  sex-specific recombination rate
chromosome homolog2:          Locus1 ----- Locus2 ----- Locus3       (rr.female, rr.male)
```

Using diploid forward simulations to track alleles at three linked autosomal loci of equal recombination distance apart, we simulated for each of 120 generations:

1. the migration of individuals into and out of each population (m=0.2*Ne individuals per generation per year with a female-biased migration ratio of 3females:1male mimicking ratios found in migrating marine fish);
2. for each individual, the production of haploid gametes via meiosis with sex-specific recombination rate among neighboring loci specified by probabilities (rr.male and rr.female)
3. the pairing of male and female gametes to produce 100 diploid offspring per breeding pair (clutch size = 100).
4. strong selection on offspring with survival dependent on the additive effects of the multilocus haplotypes carried by each individual and the habitat in which they are found (e.g. in freshwater and marine habitats selection favors the additive number of freshwater and marine alleles respectively; in intertidal populations selection favors individuals with cis-linked haplotypes of marine and/or freshwater alleles on both chromosome homologs; see Table S9 for more information)
5. finally to maintain equal population sizes, we imposed a population carrying capacity of 200 by randomly selecting 200 of the surviving offspring from all breeding pairs in the current generation (g) to continue as breeders in the subsequent generation (g+1).

We started each simulation with a two-locus secondary contact scenario where Population 1 carried freshwater adaptive alleles at Locus 1 and 3, while all other populations carried marine alleles. (Locus 2 is initially invariant among all populations, with no effect on offspring survival). We allowed migration, breeding (with recombination) and selection to occur for 30 generations during which a selection-

migration balance is quickly established and maintains divergent allele frequencies in Population 1 and 3. Even with the maintenance of this divergence, the freshwater adaptive allele spreads from population 1 through the hybrid zone into population 3 and subsequently invades the parallel hybrid zone (populations 4 and 5) with a second selection-migration balance maintaining divergence in allele frequencies between populations 3 and 5. Then, after 30 generations, a new freshwater-beneficial mutation was introduced at Locus 2, by the arrival into freshwater Population 1 of N=0.01*ne migrants homozygous for freshwater alleles at all three loci. With locus 2 no longer neutral, from this point on the survival probabilities of offspring now depended on their three locus haplotypes.

We ran 8 different simulations that differed in the extent of heterochiasmy and recombination suppression in cis-(coupled)-heterozygotes (see Table S8), and for each simulation tracked allele frequencies at each locus in each population. Each of the 8 simulations was replicated 16 times. We were specifically interested in differences in how quickly the new freshwater adaptive mutation at Locus 2 was able to establish and increase to high frequency in Population 1 (where it was first introduced), as well as how the different parameters influenced the speed at which this new allele was able to spread and establish in Population 5 (the freshwater population in the neighbouring hybrid zone; see Sup Figs 9-16). Simulations were written and run in R.

Under all simulations, recombination suppression in cis(coupled)heterozygotes significantly increases the rate at which adaptive alleles establish in the population into which they are introduced (eg. compare simulation 1 and 2), and for the most part the rate at which they spread through hybrid zones into new freshwater populations (e.g compare simulation 7 and 8). In contrast, strong heterochiasmy compared to no heterochiasmy slows the rate at which adaptive alleles establish in a population (eg. 1 vs 3), but has little effect when there is recombination suppression in cis(coupled)heterozygotes (2 vs 4). When considering heterochiasmy as a derived recombination-suppression state in males, compared to an ancestral state where males recombine equally fast as females (simulation 3 vs 5), heterochiasmy is slightly more likely to facilitate the establishment of a new allele in the population into which it was introduced, as well as the spread of that allele to other populations. However if heterochiasmy is viewed as a derived state of increased recombination in females compared to an ancestral state of low recombination in both sexes (e.g. 3 vs 7), it significantly decreases the rate at which alleles are fixed but not the rate at which they spread.

2) Custom library preparation protocol

This protocol is optimized for high-throughput preparation of genomic DNA libraries compatible for sequencing on Illumina HiSeq 3000. All DNA size selections and clean ups were performed following Ampure XP® (Beckman-Coulter) SPRI bead size selection protocol.

Step 1: DNA fragmentation using Covaris® LE220 instrument

- Dilute 300-500 ng of good quality genomic DNA in 1X TE buffer to a total volume of 130 µl and transfer to covaries 96 well microtube plate (SKU:520078)

- Insert sample filled plate into the Covaris® LE220 plate holder and run with the following settings to obtain an average fragment size of 300bp.

Sample volume	130µl
Duty factor	30%
Peak Incident Power	450
Cycles per Burst	200
Treatment time	80 sec

- Retrieve sheared samples from Covaris® plate into a 96 well plate and perform bead clean up. The following method is designed to yield fragments of size 300 bp or above.

Input DNA:	130µl
SPRI volume	104µl (0.8 times the sample volume)
Resuspension volume	30µl
Elution volume	30µl

Step 2: End repair

- Transfer 15µl of the eluate from above into a fresh plate for end repair
- Prepare the following reaction mix and add it to the DNA

**Reagent**	**1x**
Water	3.75 µl
10X T4 DNA ligase buffer	2.5 µl
10mM dNTP mix	1 µl
T4 DNA polymerase	1.25 µl
Klenow DNA polymerase	0.25 µl
T4 Polynucleotide kinase	1.25 µl
**Total volume**	**10 µl**

- Add 10 µl of reagent into 15 µl of sample. Mix well and spin down. Incubate at room temperature for 30 minutes

- Perform DNA size selection to remove fragments larger than 500 bp.

Input DNA	25 µl
SPRI volume	15 µl (0.6 times the sample volume)
PEG to save	40 µl (saving fragments < 500 bp)
SPRI volume	15 µl (add to the saved PEG to extract all smaller sized DNA)
Resuspension volume	18 µl
Final elution volume	17 µl

- Store samples in fridge if not proceeding right away

Step 3: A-tailing

- Prepare the following master mix and add it to the sample

**Reagent**	**1x µl**
NEB Buffer 2	2.5 µl
1 mM dATP	0.5 µl
Klenow exo-	0.5 µl
Water	4.5 µl
**Total**	**8 µl**

- Incubate at 37^0C for 30 minutes, then heat inactivate at 75^0C for 20 minutes
- Save 1 µl sample for running on bioanalyzer or 2 µl for checking on gel
- Directly proceed to the next step without waiting

Step 4: Adapter ligation

- Mix 1 µl 10 µM adapters with DNA sample (Illumina TruSeq single index adapter ordered from IDT)
  Note down the unique barcode used for each sample.
- Add the following ligase master mix to each sample

**Reagent**	**1x**
10mM rATP	3 µl
T4 DNA ligase	1.5 µl
Water	0.5 µl
**Total**	**5 µl**

- Incubate15 minutes at 20^0C. Then 5 minutes at 65^0C to inactivate the ligase. Allow samples to cool before proceeding

- Perform bead clean-up to remove the enzymes

DNA volume:	31 µl
SPRI volume	24.8 µl (0.8X the sample volume)
Resuspension volume	15 µl
Elution volume	14 µl

Step 5: Library enrichment by PCR

- Add the following ligase master mix to each sample

**Reagent**	**1x**
Water	2.75 µl
5x Buffer	5 µl
10mM dNTP	0.5 µl
10µM Truseq PCR1	1.25 µl
10µM Truseq PCR2	1.25 µl
Phusion polymerase	0.25 µl
**Total**	**11 µl**

- Perform PCR reaction with the following condition

95^0C	30 sec	1 cycle
98^0C	15 sec	6 cycles
62^0C	30 sec	
72^0C	30 sec	
72^0C	10 min	1 cycle
4^0C	Hold	Infinitely

Step 6: Library validation and pooling

- Confirm adapter ligation by running the sample on bio-analyzer or checking on gel (load 2 µl sample). Successful adapter ligation will increase the fragment size by ~120 bp.
- Measure final library concentration by TECAN plate reader (using picogreen dye)
- Pool equal quantity of all libraries to be sequenced in a lane
- Perform bead clean up on pooled libraries to remove PCR reagents

If pooled sample volume is less than 50 µl, make up to 50 µl by adding EB buffer

Sample volume	50 µl
SPRI volume	40 µl (0.8 times the sample volume)
Resuspension volume	61 µl
Elution volume	60 µl

- Perform DNA double size selection to remove fragments larger than 600 bp and smaller than 300 bp

Input DNA	60 µl
SPRI volume	24 µl (0.4 times the sample volume)
PEG to save	84 µl (saving fragments < 600 bp)
SPRI volume:	18 µl (add to the saved PEG to select all fragments >300 bp)
Resuspension volume	31 µl
Final elution volume	30 µl

- Check the quality of the library pools by bio-analyzer (Supplementary Figure 19) and measure the quantity by qubit high-sensitivity reagent.

- Submit 2.5nM library for sequencing

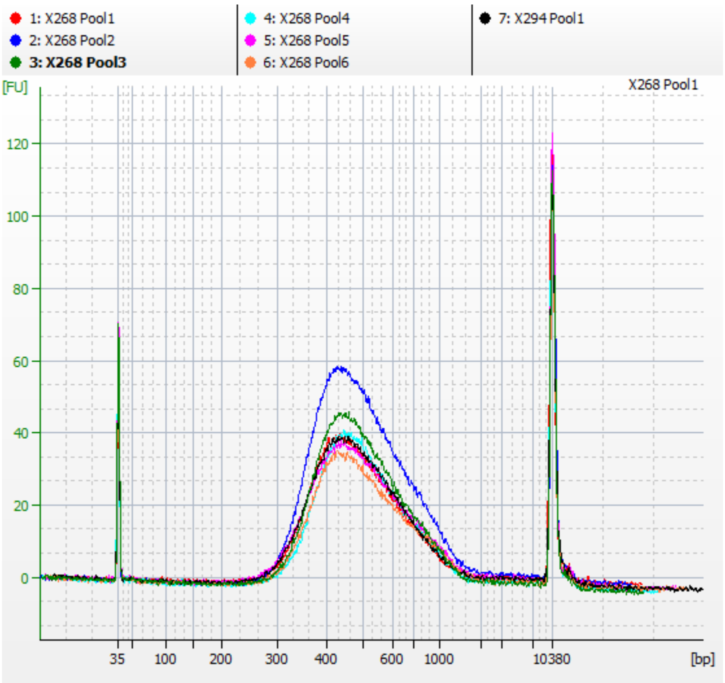

Supplementary Figure 19. *Bioanalyzer profile of size selected library pools. Library size profile of 7 pools are shown. All of them have an average fragment size of about 420 bp (300 bp insert + 120 bp adapter).*

Reagents used for library preparation:

Reagent	NEB catalogue number
T4 DNA polymerase	M0203L
T4 Polynucleotide kinase	M0201L
Klenow exo-	M0212L
T4 DNA ligase	M0202L
Klenow DNA polymerase	M0210L or M0210S
Phusion High-Fidelity DNA polymerase	M0530L

References

1 Nguyen N, Vincens P, Dufayard JF, Roest Crollius H, Louis A. Genomicus in 2022: comparative tools for thousands of genomes and reconstructed ancestors. *Nucleic Acids Research* 2022;50:D1025-D1031.

2 Urton JR, McCann SR, Peichel CL. Karyotype differentiation between two stickleback species (Gasterosteidae). *Cytogenetic and Genome Research* 2011;135:150-159

3 Varadharajan S, Rastas P, Löytynoja A, Matschiner M, Calboli F *et al*., A High-Quality Assembly of the Nine-Spined Stickleback (Pungitius pungitius) Genome. *Genome Biology and Evolution 2019;*11:3291-3308
