# Supplementary material for: Fine-scale contemporary recombination variation and its fitness consequences in adaptively diverging stickleback fish
Source: Nat Ecol Evol. 2024 Jun 5;8(7):1337–52. doi: 10.1038/s41559-024-02434-4 (PMC11239493; doi:10.1038/s41559-024-02434-4)
Supplement: Supplementary file 2 — Reporting Summary [file 41559_2024_2434_MOESM2_ESM.pdf]

nature portfolio

Corresponding author(s): Jones, Felicity C.

Last updated by author(s): 10.01.2023

Reporting Summary

Nature Portfolio wishes to improve the reproducibility of the work that we publish. This form provides structure for consistency and transparency in reporting. For further information on Nature Portfolio policies, see our Editorial Policies and the Editorial Policy Checklist.

Statistics

For all statistical analyses, confirm that the following items are present in the figure legend, table legend, main text, or Methods section.

n/a	Confirmed	
☐	☒	The exact sample size (*n*) for each experimental group/condition, given as a discrete number and unit of measurement
☐	☒	A statement on whether measurements were taken from distinct samples or whether the same sample was measured repeatedly
☐	☒	The statistical test(s) used AND whether they are one- or two-sided<br>*Only common tests should be described solely by name; describe more complex techniques in the Methods section.*
☐	☒	A description of all covariates tested
☐	☒	A description of any assumptions or corrections, such as tests of normality and adjustment for multiple comparisons
☐	☒	A full description of the statistical parameters including central tendency (e.g. means) or other basic estimates (e.g. regression coefficient) AND variation (e.g. standard deviation) or associated estimates of uncertainty (e.g. confidence intervals)
☐	☒	For null hypothesis testing, the test statistic (e.g. *F*, *t*, *r*) with confidence intervals, effect sizes, degrees of freedom and *P* value noted<br>*Give P values as exact values whenever suitable.*
☒	☐	For Bayesian analysis, information on the choice of priors and Markov chain Monte Carlo settings
☒	☐	For hierarchical and complex designs, identification of the appropriate level for tests and full reporting of outcomes
☐	☒	Estimates of effect sizes (e.g. Cohen's *d*, Pearson's *r*), indicating how they were calculated

Our web collection on statistics for biologists contains articles on many of the points above.

Software and code

Policy information about availability of computer code

Data collection	Data collection is fully described in the paper.
Data analysis	Please see code availability statement and github https://github.com/felicitycjones/Venu_SticklebackRecombination

For manuscripts utilizing custom algorithms or software that are central to the research but not yet described in published literature, software must be made available to editors and reviewers. We strongly encourage code deposition in a community repository (e.g. GitHub). See the Nature Portfolio guidelines for submitting code & software for further information.

Data

Policy information about availability of data

All manuscripts must include a data availability statement. This statement should provide the following information, where applicable:

- Accession codes, unique identifiers, or web links for publicly available datasets
- A description of any restrictions on data availability
- For clinical datasets or third party data, please ensure that the statement adheres to our policy

Fully described in the manuscript. Please see data availability statement and NCBI bioproject (PRJNA1062151) which contains:
1. short read whole genome sequencing data for each of the family pedigrees (1721 Biosamples, 3956 fastq files)
2. NCBI GEO submission superseries (GSE254561) comprising of: DMC1 chipseq files (chip & input) for both meiotic tissue (testes) and somatic tissue (liver); GEO GSE254557; H3K4me3 chipseq files (chip & input) for both meiotic tissue (testes) and somatic tissue (liver); GEO GSE254557; ATACseq data files; GEO GSE254559.

Research involving human participants, their data, or biological material

Policy information about studies with human participants or human data. See also policy information about sex, gender (identity/presentation), and sexual orientation and race, ethnicity and racism.

Reporting on sex and gender	NA
Reporting on race, ethnicity, or other socially relevant groupings	*Please specify the socially constructed or socially relevant categorization variable(s) used in your manuscript and explain why they were used. Please note that such variables should not be used as proxies for other socially constructed/relevant variables (for example, race or ethnicity should not be used as a proxy for socioeconomic status).*<br>*Provide clear definitions of the relevant terms used, how they were provided (by the participants/respondents, the researchers, or third parties), and the method(s) used to classify people into the different categories (e.g. self-report, census or administrative data, social media data, etc.)*<br>*Please provide details about how you controlled for confounding variables in your analyses.*
Population characteristics	*Describe the covariate-relevant population characteristics of the human research participants (e.g. age, genotypic information, past and current diagnosis and treatment categories). If you filled out the behavioural & social sciences study design questions and have nothing to add here, write "See above."*
Recruitment	*Describe how participants were recruited. Outline any potential self-selection bias or other biases that may be present and how these are likely to impact results.*
Ethics oversight	*Identify the organization(s) that approved the study protocol.*

Note that full information on the approval of the study protocol must also be provided in the manuscript.

Field-specific reporting

Please select the one below that is the best fit for your research. If you are not sure, read the appropriate sections before making your selection.

☒ Life sciences ☐ Behavioural & social sciences ☐ Ecological, evolutionary & environmental sciences

For a reference copy of the document with all sections, see nature.com/documents/nr-reporting-summary-flat.pdf

Life sciences study design

All studies must disclose on these points even when the disclosure is negative.

Sample size	Sample Sizes are all fully described in the paper - Nuclear Families: 18 families comprising 6 marine, 6 fresh and 6 F1hybrid intercross families, each family with 2 parents and median 93 offspring, range 86-94); ChIPseq and ATACseq: 20 individuals in metiotic (testes) and somatic tissue (liver). Hybrid Zone: 1045 individuals from 8 sites along hybrid zone of which 285 are young-of-year from within admixture zone (sites 2,3,4).
Data exclusions	Exclusions are fully described in the paper - Nuclear Families: Individual offspring were excluded if DNA extraction or library production failed. Hybrid Zone: Individuals were excluded if they had a high variant calling rate on the Illumina Golden Gate array (>=20% missing genotypes). 2265 variants were retained after those with poor clustering or high failure rate (>=20%) across all individuals.
Replication	Replication is fully described in the paper - Nuclear Families, ChIPseq and ATAC: All replicates involved biological replicates (multiple individuals in each group). No technical replicates (same individual assayed twice) were performed, though in several cases the same genomic library was sequenced in several Illumina flow cell lanes. Hybrid Zone: Due to time and resource limitations, we did not reproduce our hybrid zone study in a later year or different hybrid zone system.
Randomization	No randomization was performed.
Blinding	No blinding was performed

Reporting for specific materials, systems and methods

We require information from authors about some types of materials, experimental systems and methods used in many studies. Here, indicate whether each material, system or method listed is relevant to your study. If you are not sure if a list item applies to your research, read the appropriate section before selecting a response.

Materials & experimental systems

n/a	Involved in the study
☐	☒ Antibodies
☒	☐ Eukaryotic cell lines
☒	☐ Palaeontology and archaeology
☐	☒ Animals and other organisms
☒	☐ Clinical data
☒	☐ Dual use research of concern
☒	☐ Plants

Methods

n/a	Involved in the study
☐	☒ ChIP-seq
☐	☒ Flow cytometry
☒	☐ MRI-based neuroimaging

Antibodies

Antibodies used

H3K4me3 antibody: Rabbit polyclonal Anti-trimethyl-Histone H3 (Lys4) antibody raised against synthetic peptide from Millipore (cat#07-473)). DMC1 antibody: Guinea pig polyclonal Anti-stickleback DMC1 antibody raised against E.Coli expressed and purified stickleback DMC1 protein

Validation

Supplementary Figure 17 and MIllipore cat#07-473 (H3K4me3).

Animals and other research organisms

Policy information about studies involving animals; ARRIVE guidelines recommended for reporting animal research, and Sex and Gender in Research

Laboratory animals

Wild derived strains of marine and freshwater threespine stickleback fish Gasterosteus aculeatus collected from the River Tyne, East Lothian, Scotland and housed in stickleback facility at Max Planck Tübingen. Fish were reared under 3.5ppt salinity, with a daylight regime cycling from "winter" short (18hours dark:6hours light) to "summer" long (6hours dark:18hours long) over 180days. Fish were fed a mix of frozen, gamma irradiated, insect larvae from marine and freshwater habitats (blood worm, mysis shrimp, artemia, daphnia, cyclops). The age of individuals used for whole genome sequencing of nuclear families, ChIPseq and ATACseq is fully described in manuscript.

Wild animals

Threespine stickleback fish Gasterosteus aculeatus. Study Description of Hybrid Zone component:
Spatial and within-cohort temporal study of genotypes along a marine-freshwater hybrid zone transect in River Tyne, East Lothian, Scotland (Jones et al 2006). Sampling strategy: individuals were sampled using wire mesh minnow traps. Fin clips were taken from caudal fin, standard length measured using callipers, and fish were tagged using an elastomer tag (see Jones et al 2006) before being released back into the river. This enabled us to identify already sampled individuals (mark-recaptures) and avoid resampling. Timing and spatial scale: Sampling was carried out from Site to Site 8 on a monthly basis during 2003, see Jones et al 2006. Data collection: DNA was extracted from fincips and genotyped using a custom Illumina Golden Gate array.

Reporting on sex

Both sexes are studied. This information is provided throughout the manuscript

Field-collected samples

Please see above; DNA was extracted from fincips and genotyped using a custom Illumina Golden Gate array.

Ethics oversight

All animal experiments were done in accordance with regulations of the EU and of the state of Baden-Württemberg, Germany (Competent authority: Regierungspraesidium Tübingen, Germany; Permit and notice numbers 35/9185.82-5, 35/9185.46). Field sampling permissions were obtained from the local land owners, regional District Salmon Fisheries Board, Scottish Natural Heritage.

Note that full information on the approval of the study protocol must also be provided in the manuscript.

Plants

Seed stocks

Report on the source of all seed stocks or other plant material used. If applicable, state the seed stock centre and catalogue number. If plant specimens were collected from the field, describe the collection location, date and sampling procedures.

Novel plant genotypes

Describe the methods by which all novel plant genotypes were produced. This includes those generated by transgenic approaches, gene editing, chemical/radiation-based mutagenesis and hybridization. For transgenic lines, describe the transformation method, the number of independent lines analyzed and the generation upon which experiments were performed. For gene-edited lines, describe the editor used, the endogenous sequence targeted for editing, the targeting guide RNA sequence (if applicable) and how the editor was applied.

Authentication

Describe any authentication procedures for each seed stock used or novel genotype generated. Describe any experiments used to assess the effect of a mutation and, where applicable, how potential secondary effects (e.g. second site T-DNA insertions, mosiacism, off-target gene editing) were examined.

ChIP-seq

Data deposition

☒ Confirm that both raw and final processed data have been deposited in a public database such as GEO.

☒ Confirm that you have deposited or provided access to graph files (e.g. BED files) for the called peaks.

Data access links<br>*May remain private before publication.*	Please see NCBI BioProject PRJNA1062151, and GEO submission superseries GSE254561 (with subseries GSE254557 & GSE254559)
Files in database submission	For both DMC1 and H3K24me3 chip experiments the following files have been provided: Chip sequencing sequencing data files (fastq format) for input and chip, peak call narrowPeak files (narrowPeak) and signal track (bedgraph) files; For DMC1 ChIPseq an additional bed file of Type 1 single stranded DNA has been provide.
Genome browser session<br>(e.g. UCSC)	Genome browser tracks narrowPeak and bdg files have been provided

Methodology

Replicates	none
Sequencing depth	DMC1 ChIPseq with a mean of 33.9M read pairs; H3K4me3ChIPseq with a mean of 29.8M read pairs; ATACseq with a mean of 19.7M read pairs
Antibodies	Please see details specified above
Peak calling parameters	H3K4me3 : macs2 callpeak -t <treatment_file> -c <input_file> --SPMR -g 463000000<br>DMC1: macs2 callpeak -t <treatment_file> -c <input_file> -q 0.1 --nomodel --slocal 5000 --llocal 10000 --extsize 800 -f BED --SPMR -g 463000000 -B
Data quality	MACS2 peak calling (see paper for details) resulted in 1160 and 437 DMC1ChIPseq peaks called from type1 ssDNA fragmetns with FDR<=0.05 and signal enrichment >=5 in testes and liver respectively. 14479 and 18616 H3K4me3ChIPseq peaks with FDR<=0.05 and signal enrichment >=5 were obtained from testes of freshwater and marine strains respectively.
Software	Trimmomatic and MACS2 v2.1.1 following Khil et al (2012) and associated perl script therein.

Flow Cytometry

Plots

Confirm that:

☒ The axis labels state the marker and fluorochrome used (e.g. CD4-FITC).

☒ The axis scales are clearly visible. Include numbers along axes only for bottom left plot of group (a 'group' is an analysis of identical markers).

☒ All plots are contour plots with outliers or pseudocolor plots.

☒ A numerical value for number of cells or percentage (with statistics) is provided.

Methodology

Sample preparation	Freshly collected testes were dissociated with 0.07U/ml Liberase (Sigma) in PBS.
Instrument	BD FACSMelody Cell Sorter (BD Biosciences)
Software	BD FACSChorus™ Software (BD Biosciences)
Cell population abundance	Across six replicates Mean=3,26% of Total events with Stdev=1,81, and Mean=8,17% of Parent population (FSC singlets) with Stdev=3,8.
Gating strategy	SSC-A & FSC-A for cells; SSC-W & SSC-H for SSC singlets; FSC-W & FSC-H for FSC singlets; SSC-A & FSC-A for primary spermatocytes.

☒ Tick this box to confirm that a figure exemplifying the gating strategy is provided in the Supplementary Information.
